# Chromatic Polynomials of *F* *n*× *P*2  Graphs: Algebraic Analysis and Scheduling Applications

**DOI:** 10.12688/f1000research.176896.1

**Published:** 2026-03-04

**Authors:** Sarah M. Talab, Nabeel E. Arif

**Affiliations:** 1 Mathematics, Tikrit University, Tikrit, Saladin Governorate, 34001, Iraq

**Keywords:** Graph coloring, Chromatic polynomial, Cartesian product, Friendship graph, Combinatorial mathematics, Recurrence relation, Closed-form expression, Scheduling.

## Abstract

**Background:**

Chromatic polynomials are fundamental algebraic invariants in graph theory, bridging pure mathematics and practical applications. While extensive results exist for paths and cycles, the Cartesian product $F_{n}\times P_{2}$ remains largely unexplored despite its layered constraint structure, presenting a clear gap in the literature.

**Methods:**

We employ combinatorial decomposition and recursive block construction, applying the inclusion–exclusion principle to the eight edge constraints within each recursive unit. This analytical approach enables the derivation of the chromatic transition polynomial ψ(k) , which governs the recurrence relations and closed-form expressions.

**Results:**

We establish the recurrence relation $P_{n}\left(k\right)=\psi \left(k\right)\;P_{n-1}\left(k\right)$ and the closed-form expression $P_{n}\left(k\right)={\left[\psi \left(k\right)\right]}^{n-2}\;P_{2}\left(k\right)$ where $\psi \left(k\right)=k^{4}-8k^{3}+26k^{2}-41k+26.$ The chromatic number is proven to be χ=3 , with real roots of ψ(k) located within [2,3]. Numerical validation confirms both recurrence and closed-form formulas, while asymptotic analysis shows the exponential growth of $P_{n}\left(k\right)\;$ is governed by ψ(k) , as $\lim_{n\to \infty }{\left[P_{n}\left(k\right)\right]}^{\frac{1}{n}}=\left|\psi \left(k\right)\right|$ .

**Conclusions:**

This research provides a comprehensive algebraic characterization of the chromatic polynomial for $F_{n}\times P_{2}$ , deriving its recurrence relation and closed-form expression. Building on this foundation, we develop a novel two-period conference scheduling model where the chromatic polynomial serves as a quantitative tool to compute all conflict-free room allocations. This work demonstrates directly how structural graph theory can inform practical resource allocation systems, transforming an abstract invariant into a concrete decision-support tool.

## 1. Introduction

Chromatic polynomials are considered a fundamental tool in algebraic graph theory. Initially introduced by Birkhoff (1912) in his attempt to prove the four-color conjecture, their development was profoundly advanced by Whitney’s (1932) deletion-contraction recurrence and Read’s (1968) systematic studies, culminating in a comprehensive review by Tutte and Read (1988).

The chromatic polynomial P(G,k) , which counts the proper k -colorings of a graph G , also bears significant practical importance. It serves as a critical tool in diverse applied fields, including task scheduling, ^1^ data analysis, ^2^ network design, ^3^ theoretical chemistry, ^4^ and statistical physics. ^5^ However, computing the chromatic polynomial remains a challenging problem, particularly for graphs constructed from Cartesian products (G×H) , where determining a general formula relating P(G×H,k) to its factor polynomials is NP -hard. This challenge has encouraged the derivation of closed-form expressions for specific graph classes.

The properties of Cartesian products, including their coloring characteristics, have provided a solid theoretical basis for studying composite graphs. ^6^ The effect of these graph operations on coloring and dominance has been widely investigated as a key to understanding the structure of composite graphs. ^7^ Notably, recent studies have successfully derived chromatic polynomials for other composite structures, such as the Triangular Snake and the n-Centipede graphs using structural recursion. ^8^

While significant research has focused on Cartesian products of basic graphs, such as paths ( $P_{n}$ ) and cycles( $C_{n}$ ), ^9– 12^ the structure $F_{n}\times P_{2}$ —formed by the Cartesian product of a friendship graph and a 2-path—has received little attention. Its unique composition, which interlaces local triangular clusters with a linear, two-layer framework, presents a compelling subject for algebraic graph theory.

This paper provides a complete analytical framework for $F_{n}\times P_{2}$ by:

• Deriving its recurrence relation and a closed-form expression for its chromatic polynomial.
• Establishing its chromatic number, analyzing the root distribution of its transition polynomial, and determining its asymptotic growth rate.
• Validating the theoretical results through numerical computation.
• Demonstrating its practical utility via a novel application to a two-period conference scheduling problem.

This practical approach aligns with recent results ^1, 13^ that confirm the utility of graph coloring in modeling scheduling constraints and resolving resource conflicts, thereby reinforcing the practical relevance of our findings.

## 2. Preliminaries

**Figure 1.**
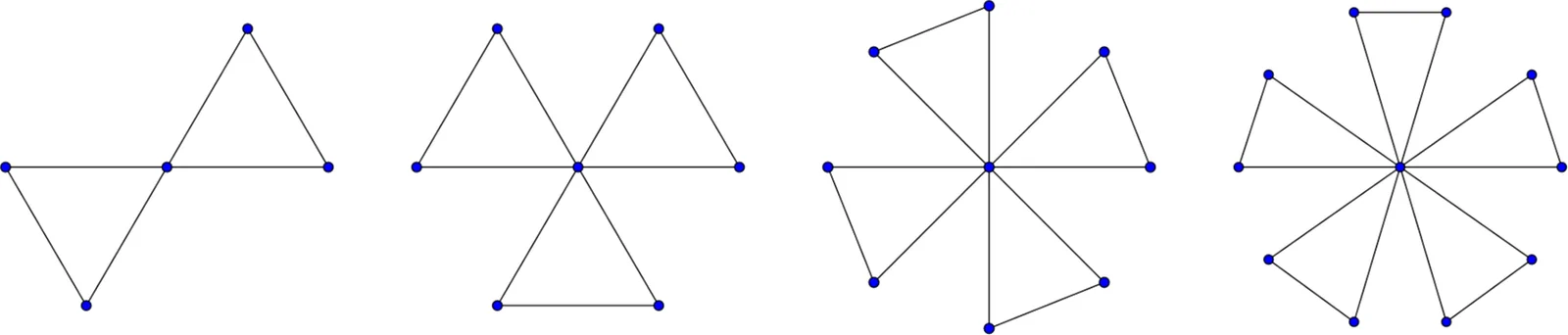
Friendship graphs $F_{n}$ for n=2,3,4,5. Each graph is formed by n triangles sharing a common central vertex $v_{0}$ , illustrating the recursive structure of the friendship graph family.

Definition 2.1 ^14^: A friendship graph, denotes $F_{n}\;$ for n≥2 , and it’s defined as the union of n copies of cycles $\left(C_{3}\right)$ with a common vertex (the center). Formally:

• Vertex set: $V\left(F_{n}\right)=\left\{v_{0}\right\}\cup {\left\{u_{i},w_{i}\right\}}_{i=1}^{n}$ where $v_{0}$ is the center vertex.
• Edge set: $E\left(F_{n}\right)={\left\{(v_{0},u_{i}),(v_{0},w_{i}),(u_{i},w_{i})\right\}}_{i=1}^{n}$ .
• Order: $|V\left(F_{n}\right)|=2n+1$ .
• Size: $|E\left(F_{n}\right)|=3n$ .
Figure 1 (Friendship graphs *F*_*n*_):

Definition 2.2 ^6^: The Cartesian product of two graphs G and H denotes (G×H) and it’s known as a graph in which each vertex is an ordered pair (u,w) where u∈V(G)andw∈V(H) , creating an edge between two vertices $\left(u_{1},w_{1}\right)$ and $\;\left(u_{2},w_{2}\right)$ .

if:

•
$u_{1}=u_{2}\;$ and $w_{1}$ is adjacent to $w_{2}$ in H , or
•
$w_{1}=w_{2}$ and $u_{1}$ is adjacent to $u_{2}$ in G .

Definition 2.3 ^15^: The chromatic polynomial P(G,k) is a polynomial in k that expresses the number of proper vertex k -colorings of G , such that adjacent vertices share distinct colors.

Definition 2.4: The graph $F_{n}\times P_{2}$ is the Cartesian product of a friendship graph $\;F_{n}$ with a path $P_{2}$ , forming two parallel layers of $\;F_{n}$ with corresponding vertices connected by edges.

**Figure 2.**
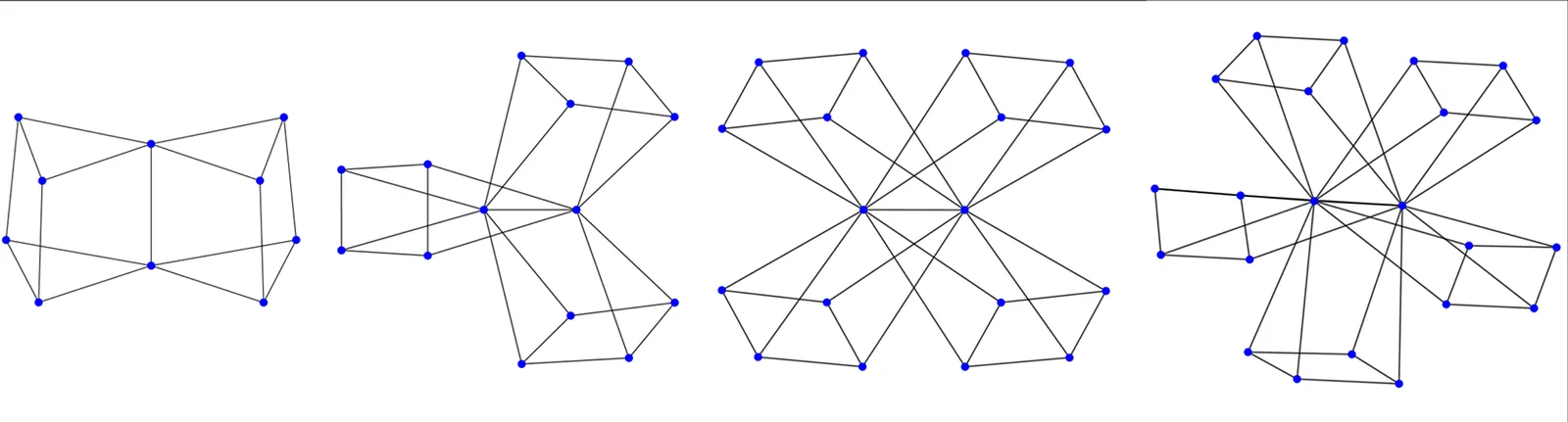
Structure of the Cartesian Product $F_{n}\times P_{2}$ for n=2,3,4,5. This construction yields two parallel layers of $F_{n}$ , with corresponding vertices connected by vertical edges.

Figure 2 (Cartesian product *F*_*n*_ × *P*_2_):

## 3. Methods

### 3.1 Analytical framework and structural decomposition

This is a theoretical study in algebraic and combinatorial graph theory, analyzing the chromatic polynomial of the graph family $G_{n}=F_{n}\times P_{2}$ . The core of our approach is a structural decomposition that reveals a recursive construction. For n≥3 , $G_{n}$ is obtained from $G_{n-1}$ by attaching a new block $B_{n}$ .This block contains four new vertices $\left\{u_{n}^{A},w_{n}^{A},u_{n}^{B},w_{n}^{B}\right\}$ and eight edges that form two new triangles (one in each layer) along with their vertical connections. Crucially, $B_{n}$ attaches only to the two central vertices $v_{0}^{A}$ and $v_{0}^{B}$ of $G_{n-1}$ . This localized, exclusive attachment is the key to isolating the chromatic contribution of each step.

### 3.2 Combinatorial derivation of the transition polynomial ψ(k)

The proper coloring of the new block $B_{n}$ depends solely on the colors assigned to its two attachment points, $v_{0}^{A}$ and $v_{0}^{B}$ , which must be distinct in any proper coloring of the base graph $G_{n-1}$ . We compute the number of proper k -colorings of $B_{n}$ under this condition, denoted $N_{\text{diff}}\left(k\right)$ , using the inclusion-exclusion principle applied to its eight edge constraints.

Let S be the set of all colorings of the four new vertices without constraints, so $|S|=k^{4}$ . For an edge $e_{i}$ , let $M_{i}$ be the set of colorings where its endpoints share the same color. Then:

$$N_{\text{diff}}\left(k\right)=\left|S\right|-\sum_{i=1}^{8}\left|M_{i}\right|+\sum_{1\;\leq \;i<j\;\leq \;8}\left|M_{i}\;\cap \;M_{j}\right|-\sum_{1\;\leq \;i<j<t\;\leq \;8}\left|M_{i}\;\cap \;M_{j}\;\cap \;M_{t}\right|+\ldots +{\left(-1\right)}^{8}\left|M_{1}\;\cap \;M_{2}\;\cap \;\ldots \;\cap \;M_{8}\right|$$

**Coefficient Analysis:**

$-8k^{3}$ : Each of the 8 edges defines a single constraint $\left|M_{i}\right|=k^{3}$ , because fixing the color of one endpoint (or satisfying the equality) leaves 3 vertices free.

$+26k^{2}$ : There are 26 compatible edge pairs (out of 28) that can be satisfied simultaneously without color conflicts under the distinct‑central‑colors condition, each giving $\left|M_{i}\;\cap \;M_{j}\right|=k^{2}$ .

−41k : Analysis of compatible edge triples yields 41 configurations with $\left|M_{i}\;\cap \;M_{j}\;\cap \;M_{t}\right|=k$ .

+26 : The full intersection of all eight constraints corresponds to 26 distinct colorings consistent with the distinct central colors.

This combinatorial enumeration yields the chromatic transition polynomial:

$$N_{\text{diff}}\left(k\right)=k^{4}-8k^{3}+26k^{2}-41k+26=\psi \left(k\right)$$

This result is algebraically verified by exact polynomial division: $\psi \left(k\right)=\;\frac{P_{3}\left(k\right)}{P_{2}\left(k\right)}$ for all k≥3.

Remark 3.2.1 (Methodological Soundness)

The polynomial ψ(k) is validated through dual independent approaches:

The combinatorial inclusion–exclusion derivation provides structural insight into the constraint system, while the exact polynomial division $\left(\psi \left(k\right)=\;\frac{P_{3}\left(k\right)}{P_{2}\left(k\right)}\right)$ offers algebraic confirmation, ensuring mathematical rigor.

### 3.3 Derivation of the recurrence and closed-form expression

The structural isolation of $B_{n}$ implies that any proper coloring of $G_{n}$ can be obtained by independently choosing:

(i) a proper coloring of $G_{n-1}$ and
(ii) a proper coloring of $B_{n}$ consistent with the colors of the central vertices in $G_{n-1}$ . Consequently, the chromatic polynomials satisfy the fundamental recurrence relation for n≥3 :

$$P_{n}\left(k\right)=N_{\text{diff}}\left(k\right)\;P_{n-1}\left(k\right)=\psi \left(k\right)\;P_{n-1}\left(k\right)$$

Applying mathematical induction to this recurrence, with $P_{2}\left(k\right)$ as the verified base case, provides the closed-form expression for all n≥2 :

$$P_{n}\left(k\right)={\left[\psi \left(k\right)\right]}^{n-2}\;P_{2}(k).$$

### 3.4 Additional analytical and numerical validation

• Chromatic Number: Combinatorial reasoning (the presence of disjoint triangles and an explicit constructive 3-coloring) establishes χ=3 .
• Root Analysis: Using calculus (evaluation of ψ(k) and its derivative, the Intermediate Value Theorem), we prove the real roots of ψ(k) lie within the interval [2,3] .
• Asymptotic Growth Rate: The closed-form expression directly implies the exponential growth rate: $\lim_{n\to \infty }{\left[P_{n}\left(k\right)\right]}^{\frac{1}{n}}=\left|\psi \left(k\right)\right|$ .
• Numerical Validation: All derived formulas are validated for integer values k≥3 using Wolfram Mathematica confirming the consistency of the recurrence and closed-form expression with directly computed values of $P_{n}\left(k\right)$ .

## 4. Results

### 4.1 Structural analysis of $F_{n}\times P_{2}$

Theorem 4.1.1: (Structural Properties and Chromatic Implications)

For n≥2 , the graph $F_{n}\times P_{2}$ possesses the following structural properties, which directly affect its chromatic behavior:

1.
|V|=4n+2.

2.
|E|=8n+1 .
3. Degree sequence $\left\{{\left(2n+1\right)}^{\left(2\right)},\;3^{\left(4n\right)}\right\}$ .

Where the notation $\;d^{\left(\eta \right)}$ denotes (η) vertices of degree d .

Proof:

1. Vertex count: Every $F_{n}$ has (2n+1) vertices. The product with $P_{2}$ resulting in 2×(2n+1)=4n+2 vertices. This layering allows a recursive coloring process.
2. Edge count consists of:
  • Internal edges: 2×3n=6n ,
  • Vertical edges: 2n (connecting peripherals) + 1 (connecting centers) = 2n+1 ,
  We get: 6n+(2n+1)=8n+1 .
3. Degree analysis:
  • Central Vertices ( 2 vertices): Every center is connected to:
    -
2n peripheral vertices in its layer.
    - 1 center vertex in the opposite layer.

deg=2n+1.

• Peripheral Vertices ( 4n vertices): Every peripheral vertex is connected to:
  - 1 center vertex in its layer.
  - 1 partner vertex in the same triangle.
  - 1 opposite vertex in the opposite layer.
deg=3.

Thus, the degree sequence is $\left\{{\left(2n+1\right)}^{\left(2\right)},\;3^{\left(4n\right)}\right\}$ . ∎

**Chromatic Significance:**

This structure includes a hierarchical constraint system:

• Central vertices act as chromatic regulators, helping with color separation in both layers.
• Peripheral vertices form recursive units, which have local coloring constraints.
• Horizontal edges uphold proper coloring across time periods.
• Vertical edges prevent color reuse across sequential periods.

Theorem 4.1.2: (Edge classification and Distribution)

The edge classification derives directly from the vertex degree analysis in Theorem 4.1.1 ( Table 1).

Proof: The three edge types and their counts are determined as follows:

1. Central Edge: Exactly one edge connects the two central vertices, each of degree ( 2n+1) .
2. Center-Peripheral Edges: Each central vertex is adjacent to 2n peripheral vertices in its own layer, yielding 2×2n=4n edges of this type.
3. Uniform Peripheral Edges: This set comprises:
  • The base edges of the *n* triangles in each layer: 2×n=2n edges.
  • The vertical edges connecting corresponding peripheral vertices across layers: 2n edges.

Their total is 2n+2n=4n edges.

This completes the classification. ∎

Proposition 4.1.3: (Graph Properties)

The graph $F_{n}\times P_{2}$ is:

1. Connected, for n≥2 .
2. Non-planar for n≥3 .

Proof:

1. Since $F_{n}$ is connected, the Cartesian product with *P*_2_ adds a second layer that is linked to the first through vertical edges between corresponding vertices. These vertical connections ensure that the two layers are joined, so the resulting graph *F*_*n*_ × *P*_2_ remains connected.
2. For n≥3 , it contains ${Κ}_{3,3}$ minor; therefore, by Kuratowski’s theorem, it is non-planar. ∎

Lemma 4.1.4: (Isolation Lemma for the Block $B_{n}$ )

In the recursive construction of $G_{n}=F_{n}\times P_{2}$ ( Section 3.1), the coloring of the newly attached block $B_{n}$ is conditionally independent of the coloring of the subgraph $G_{n-1}$ . Formally, given any fixed pair of distinct colors assigned to the central vertices $v_{0}^{A}$ and $v_{0}^{B}$ , the number of valid extensions to color the four vertices of $B_{n}$ is a well-defined function $N_{\text{diff}}\left(k\right)$ that depends only on the number of colors k .

Proof: The block $B_{n}$ is adjacent to $G_{n-1}$ only at the vertices $v_{0}^{A}$ and $v_{0}^{B}$ . No edge connects $B_{n}$ to any other vertex of $G_{n-1}$ . Therefore, once colors for $v_{0}^{A}$ and $v_{0}^{B}$ are fixed, the coloring constraints are entirely contained within $B_{n}$ , making the extension count independent of the rest of $G_{n-1}$ . The symmetry of the coloring problem under permutations of the color set ensures that this count depends only on k . ∎

**Table 1.** Edge classification in $F_{n}\times P_{2}$ .

Edge type	Description	Degree of Endpoints	Count
Central Edge	Connects the two central vertices	(2n+1,2n+1)	1
Center-Peripheral Edges	Connects central to peripheral vertices	(2n+1,3)	4n
Uniform Peripheral Edges	Connects degree- 3 vertices	(3,3)	4n

### 4.2 Chromatic polynomial analysis

Theorem 4.2.1: (Recurrence Relation)

For all n≥3 , the chromatic polynomial of $F_{n}\times P_{2}$ satisfies a first-order linear recurrence governed by the chromatic transition polynomial ψ(k) :

$$P_{n}\left(k\right)=\psi \left(k\right)\;P_{n-1}\left(k\right),$$

where $\psi \left(k\right)=k^{4}-8k^{3}+26k^{2}-41k+26.$

Proof: The factorization $P_{n}\left(k\right)=N_{\text{diff}}\left(k\right)\;P_{n-1}\left(k\right)$ follows from the conditional independence in the recursive construction ( Lemma 4.1.4). The equality $N_{\text{diff}}\left(k\right)=\psi \left(k\right)$ is the result of the combinatorial enumeration detailed in Section 3.2. ∎

Theorem 4.2.2: (Closed-Form Expression)

For n≥2 , the chromatic polynomial of $F_{n}\times P_{2}$ is given by the closed-form expression:

$$P_{n}\left(k\right)={\left[\psi \left(k\right)\right]}^{n-2}\;P_{2}\left(k\right).$$

Proof: By using mathematical induction on n :

Base case (n=2) : Trivially, $P_{2}\left(k\right)={\left[\psi \left(k\right)\right]}^{0}\;P_{2}\left(k\right)$ .

Suppose the formula holds for n−1 , i.e.,

$$P_{n-1}\left(k\right)={\left[\psi \left(k\right)\right]}^{n-3}\;P_{2}\left(k\right)$$

using the recurrence relation $\;P_{n}\left(k\right)=\psi \left(k\right)\;P_{n-1}\left(k\right),$ we substitute the inductive hypothesis:

$$P_{n}\left(k\right)=\psi \left(k\right)\;\left({\left[\psi \left(k\right)\right]}^{n-3}\;P_{2}\left(k\right)\right)={\left[\psi \left(k\right)\right]}^{n-2}\;P_{2}\left(k\right).$$

∎

Corollary 4.2.3 (Computational Efficiency)

The closed-form expression $P_{n}\left(k\right)={\left[\psi \left(k\right)\right]}^{n-2}\;P_{2}\left(k\right)$ . reduces the time complexity of computing $P_{n}\left(k\right)$ from exponential (via the deletion–contraction algorithm) to O(logn) using exponentiation by squaring, providing a significant computational advantage for large n .

Proposition 4.2.4 (Chromatic Number)

For all n≥2 ,

$$\chi \left(F_{n}\times P_{2}\right)=3$$

Proof: Since $F_{n}\times P_{2}$ contains triangles (copies of $C_{3}$ ), at least 3 colors are required, establishing the lower bound χ≥3 . To show that 3 colors suffice, we construct an explicit proper 3-coloring c:V→{1,2,3} . Color the two center vertices as $c\left(v_{0}^{A}\right)=1$ and $c\left(v_{0}^{B}\right)=2$ . For each triangle i(i=1,…,n) , assign colors to the peripheral vertices as follows: $c\left(u_{i}^{A}\right)=2,\;c\left(w_{i}^{A}\right)=3$ , $c\left(u_{i}^{B}\right)=3,\;c\left(w_{i}^{B}\right)=1$ . One may verify that all edges within triangles, between centers, and vertical edges between layers receive distinct colors at their endpoints. Thus, c is a proper 3-coloring, proving the upper bound χ≤3 . Therefore, χ=3 .∎

### 4.3 Algebraic and asymptotic analysis

Theorem 4.3.1 (Real Roots of ψ(k))

The chromatic transition polynomial $\psi \left(k\right)=k^{4}-8k^{3}+26k^{2}-41k+26$ has exactly two real roots, both in the interval [2,3].

Proof: Direct evaluation gives ψ(2)=0 , ψ(2.5)=0.0625>0 , ψ(3)=2>0 .

The derivative $\psi^{\prime }\left(k\right)=4k^{3}-24k^{2}+52k-41$ satisfies $\;\psi^{\prime }\left(2\right)=-1<0\;$ , $\;\psi^{\prime }\left(2.5\right)=1.5>0$ .

**Existence:** Since $\psi^{\prime }\left(2\right)<0,\;\psi$ is decreasing at k=2 , so ψ(2+ε)<0 for small ε>0 . With ψ(2.5)>0 and ψ continuous, the Intermediate Value Theorem gives a root in (2,2.5) .

**Uniqueness:** The second derivative $\psi^{\prime \prime }\left(k\right)=12k^{2}-48k+52$ has discriminant −192<0 and positive leading coefficient, so $\;\psi^{\prime \prime }>0$ everywhere. Thus $\psi^{\prime }$ is strictly increasing and changes sign exactly once in (2,2.5), giving at most one root. Combined with existence, the root is unique.

Hence ψ has roots at k=2 and in (2,2.5) . By the Fundamental Theorem of Algebra, the remaining two roots are complex conjugates. ∎

Theorem 4.3.2 (Exponential Growth Rate)

For all k such that ψ(k)≠0 and $P_{2}\left(k\right)\neq 0$ , we have

$$\lim_{n\to \infty }{\left[P_{n}\left(k\right)\right]}^{\frac{1}{n}}=|\psi \left(k\right)|$$

Proof: From the closed-form expression in Theorem 4.2.2,

$$P_{n}\left(k\right)={\left[\psi \left(k\right)\right]}^{n-2}\;P_{2}\left(k\right)\;\left(n\;\geq \;2\right).$$

Write ψ(k) in polar form:

$$\psi \left(k\right)=|\psi \left(k\right)|e^{\mathrm{i\theta}},\;\theta =\arg\left(\psi \left(k\right)\right).$$

Then:

$${\left[P_{n}\left(k\right)\right]}^{\frac{1}{n}}={\left|\;{\left[\psi \left(k\right)\right]}^{n-2}\;P_{2}\left(k\right)\right|}^{\frac{1}{n}}={\left|\psi \left(k\right)\right|}^{1-\frac{2}{n}}\;.{\left|P_{2}\left(k\right)\right|}^{\frac{1}{n}}\cdot e^{\mathrm{i\theta}\left(1-\frac{2}{n}\right)}$$

Taking limits:

•
$\lim_{n\to \infty }\;{\left|\psi \left(k\right)\right|}^{1-\frac{2}{n}}=|\psi \left(k\right)|$ ,

because |ψ(k)| is constant and $1-\frac{2}{n}\to 1$ .

•
$\lim_{n\to \infty }{\left[P_{2}\left(k\right)\right]}^{\frac{1}{n}}=1$ ,

since $P_{2}\left(k\right)$ is a non‑zero constant polynomial, and for any constant c>0 , $c^{\frac{1}{n}}\to 1$ .

•
$|e^{\mathrm{i\theta}\left(1-\frac{2}{n}\right)}|=1$ for all n ,

since $|e^{\mathrm{i\phi}}|=1$ for every real ϕ

(here $\phi =\theta \left(1-\frac{2}{n}\right)$ ).

Therefore:

$$\lim_{n\to \infty }{\left[P_{n}\left(k\right)\right]}^{\frac{1}{n}}=|\psi \left(k\right)|.$$

∎

**Note:** For integer k≥3 , Table 2 shows ψ(k)>0 , hence in that case |ψ(k)|=ψ(k) .

Corollary 4.3.3 (Ratio Convergence of Chromatic Polynomials)

For all k such that ψ(k)≠0 , $\lim_{n\to \infty }\frac{P_{n}\left(k\right)}{P_{n-1}\left(k\right)}=\psi \left(k\right)$ .

Proof: This follows immediately from the recurrence relation

$P_{n}\left(k\right)=\psi \left(k\right)\;P_{n-1}\left(k\right)$ in Theorem 4.2.1.

For n≥3 , we have $\frac{P_{n}\left(k\right)}{P_{n-1}\left(k\right)}=\psi \left(k\right)$ ,

so the limit as n→∞ is trivially ψ(k) . ∎

**Table 2.** Numerical values of $P_{n}\left(k\right)$ for =2,3,4,and5 , along with ψ(k) at integer values k≥3 .

k	ψ(k)	$P_{2}\left(k\right)$	$P_{3}\left(k\right)$	$P_{4}\left(k\right)$	$P_{5}\left(k\right)$
3	2	24	48	96	192
4	22	5,808	127,776	2,811,072	61,843,584
5	96	184,320	17,694,720	1,698,693,120	163,074,539,520
6	284	2,419,680	687,189,120	195,161,710,080	55,425,925,662,720

**Interpretation.** The value |ψ(k)| serves as the exponential growth constant for the sequence $\left\{P_{n}\left(k\right)\right\}$ . This establishes ψ(k) as the fundamental scaling factor governing the asymptotic expansion of chromatic polynomials. For large n , $P_{n}\left(k\right)$ scales approximately as ${\left|\psi \left(k\right)\right|}^{n}$ , indicating that each increment in *n* multiplies the number of proper colorings by approximately |ψ(k)| .

Remark 4.3.4 (Asymptotic Behavior and Root Distribution)

1. Theorem 4.3.2 establishes |ψ(k)| as the exponential growth constant for the sequence $P_{n}\left(k\right)$ . For k≥3,ψ(k) itself serves this role.
2. The roots of $P_{n}\left(k\right)$ comprise:
  • The fixed roots of $P_{2}\left(k\right)$ , and
  • The roots of ψ(k) , where each root of ψ(k) has an algebraic multiplicity of n−2 .

As n→∞ , the roots of ψ(k) dominate the overall distribution, acting as accumulation points in the complex plane.

### 4.4 Numerical validation

To verify the recurrence relation $P_{n}\left(k\right)=\psi \left(k\right)\;P_{n-1}\left(k\right)$ ( Theorem 4.2.1) and its closed-form expression $P_{n}\left(k\right)={\left[\psi \left(k\right)\right]}^{n-2}\;P_{2}\left(k\right)$ ( Theorem 4.2.2), we computed the chromatic polynomials $P_{n}\left(k\right)$ for n=2,3,4,5 using Wolfram Mathematica.

Extensive verification for integer values k≥3 showed perfect agreement among three independent approaches:

1. Direct computation of $P_{n}\left(k\right)$ ,
2. Evaluation using the recurrence relation,
3. Evaluation using the closed-form expression.

This numerical validation confirms the consistency of our theoretical results, as summarized in Table 2.

Specific calculations that demonstrate the application of the derived formulas are as follows:

• Theorem 4.2.1: $P_{3}\left(3\right)=\psi \left(3\right).P_{2}\left(3\right)=2\;.\;24=48$ .
• Theorem 4.2.2: $P_{5}\left(6\right)={\left[\psi \left(6\right)\right]}^{3}\;P_{2}\left(6\right)$ $=284^{3}.\;2419680=55425925662720$ .
• Exponential growth: ${\left[P_{5}\left(4\right)\right]}^{\frac{1}{5}}={\left(61843584\right)}^{\frac{1}{5}}\;\approx \;22.000\;\approx \;\psi \left(4\right)$ .

The explicit polynomial expressions used as the basis for these computations are:

$$P_{2}\left(k\right)=k^{10}-17k^{9}+132k^{8}-614k^{7}+1882k^{6}-3932k^{5}+5581k^{4}-5165k^{3}+2808k^{2}-676k.$$

$$P_{3}\left(k\right)=k^{14}-25k^{13}+294k^{12}-2153k^{11}+10949k^{10}-40806k^{9}+114575k^{8}-245171k^{7}+399378k^{6}-488483k^{5}+435287k^{4}-266994k^{3}+100724k^{2}-17576k.$$

These computations offer conclusive verification of Theorem 4.2.1 and Theorem 4.2.2 for the checked values. Furthermore, the convergence of ${\left[P_{n}\left(k\right)\right]}^{\frac{1}{n}}$ to ψ(k) , numerically validates the exponential growth behavior established in Theorem 4.3.2.

### 4.5 Application: A chromatic model for hierarchical conference scheduling

The chromatic polynomial $P_{n}\left(k\right)$ of the graph $G=F_{n}\times P_{2}$ is used to model a constrained two-period conference scheduling system.

**4.5.1 Model Specification: A Two-Period Conference**

The conference structure is modeled by the friendship graph $F_{n}$ , where:

• Central vertex: Refers to the conference coordinator.
• Triangles: Refers to the research teams and their relationship to the coordinator.

Each team must complete two different tasks:

a. Project presentation.
b. Brainstorming and discussion.

These tasks are scheduled across two consecutive periods (e.g., morning and afternoon sessions). The system specifications include:

• Participants: One coordinator and n independent teams.
• Sessions: Two time periods with two task types per team.
• Resources: k identical meeting rooms.
• Objective: Assign rooms to all sessions while satisfying scheduling constraints.

**4.5.2 Graph-Theoretic Representation**

The graph $G=F_{n}\times P_{2}$ yield all scheduling constraints by its vertex and edge structure:

• Vertices: Refer to all sessions (coordinator and teams across both periods)
• Edges: Represent conflicts and constraints:
  - Horizontal edges: Prevent simultaneous room usage for related sessions.
  - Vertical edges: Prevent room reuse by the same team across consecutive periods.

**4.5.3 Chromatic Polynomial as Scheduling Tool**

The Chromatic polynomial $P_{n}\left(k\right)$ computes all valid room allocation schedules that satisfy the following critical conditions:

1. Conflict avoidance: No two conflicting sessions share the same room.
2. Temporal separation: No room reuse for the same team across consecutive periods.
3. Constraint compliance: All session-specific scheduling constraints are observed.

**4.5.4 Analytical Planning Insights**

The closed-form expression $P_{n}\left(k\right)={\left[\psi \left(k\right)\right]}^{n-2}\;P_{2}\left(k\right)$ offers important insights for conference planning:

• Feasibility Threshold: The real root of ψ(k) at $k_{\min}\;\approx \;2.5$ refers to the theoretical minimum rooms required, but the chromatic number $\chi \left(F_{n}\times P_{2}\right)=3$ shows the practical minimum, meaning that 3 rooms are enough for any conference size.
• Scalability Analysis: The growth rate determined by ψ(k) predicts how room demand scales with the increase in teams, establishing the fundamental mathematical relationship between conference size and resource requirements.
• Flexibility Quantification: The chromatic polynomial value $P_{n}\left(k\right)$ directly measures the theoretical flexibility space accessible to planners, with higher values indicating greater resilience against scheduling constraints.

These theoretical insights define the basic boundaries with possibilities of the scheduling system, which we will quantitatively check in the following section.

**4.5.5 Quantitative Performance Analysis**

Based on the theoretical framework, we conduct a performance analysis of the conference scheduling model using the calculated values of $P_{n}\left(k\right)$ . This translation turns abstract graph-theoretic concepts into concrete planning metrics. Our analysis focuses on three key performance measures derived from the chromatic model: (1) the practical feasibility threshold, validated by the chromatic number χ=3 ; (2) the measured scheduling flexibility, quantified directly by the chromatic polynomial $P_{n}\left(k\right)$ ; and (3) the observed scalability indicator, defined by the growth rate of $P_{n}\left(k\right)$ with respect to n .

**Scheduling with the Minimum Required Rooms (** k=3 **)**

The operational feasibility of the theoretical chromatic number is demonstrated numerically. For a growing number of teams n , the number of valid conflict-free schedules $P_{n}\left(3\right)$ exhibits exact exponential growth, doubling with each added team: $P_{n}\left(3\right)=2.P_{n-1}\left(3\right)$ . The specific values for conferences of different scales are compiled in Table 3, confirming that any conference with n≥2 teams can be scheduled with only three rooms.

**Table 3.** Conference scheduling with minimum rooms.

Conference scale	Teams ( *n*)	Valid schedules $\left(P_{n}\left(3\right)\right)$	Growth factor
Small Conference	3	48	---
Medium Conference	4	96	×2
Medium Conference	5	192	×2
Large Conference	6	384	×2

**Quantifying the Impact of Additional Resources**

To evaluate the gains from increased resources, we compare the scheduling flexibility $P_{n}\left(k\right)$ for k=3,4,5 . The results, detailed in Table 4, reveal the principle of exponential resource leverage. A single additional room (moving from k=3 to k=4 ) increases the number of valid schedules for 5 teams from 192 to over 61.8 million. This represents a gain factor of approximately 22 , which equals ψ(4) . Similarly, providing five rooms yields a gain factor of approximately 96, equaling ψ(5) . These values were computed using the closed-form expression $P_{n}\left(k\right)={\left[\psi \left(k\right)\right]}^{n-2}\;P_{2}\left(k\right)$ , where ψ(4)=22 and ψ(5)=96 .

**Table 4.** Impact of room availability on scheduling flexibility.

Scenario	k	$P_{4}\left(k\right)$	$P_{5}\left(k\right)$	Gain $\left(P_{5}\left(k\right)\;\mathrm{vs}\;P_{4}\left(k\right)\right)$
Minimal Rooms	3	96	192	×2
Added Flexibility	4	2,811,072	61,843,584	≈ ×22
High Flexibility	5	1,698,693,120	163,074,539,520	≈×96

**Key Findings**

1. Operational Feasibility: Empirical data confirm that the theoretical chromatic minimum of three rooms (χ=3) is both necessary and sufficient for practical scheduling, regardless of conference size (n≥2) .
2. Exponential Flexibility Growth: Using only the minimum rooms, scheduling flexibility grows exponentially. Each additional team doubles the number of valid schedules, as dictated by the exact recurrence $P_{n}\left(3\right)=2.P_{n-1}\left(3\right)$ , which is demonstrated by the data in Table 3.
3. Exponential Resource Leverage: Marginal increases in resources yield disproportionately large gains in flexibility. Crucially, the gain factor achieved by adding one room is exactly the value of the chromatic transition polynomial ψ(k) , directly translating a graph-theoretic invariant into a concrete measure of operational efficiency, as quantified in Table 4.

**4.5.6 Model Interpretation for Decision Support**

This framework translates the mathematical constructs of $F_{n}\times P_{2}$ and its chromatic polynomial into actionable insights for conference planners. Table 5 provides the complete, systematic mapping that underpins this translation, linking each graph-theoretic element to its concrete scheduling counterpart and its direct practical significance for decision-makers.

**Table 5.** From graph elements to scheduling decisions.

Mathematical element	Scheduling representation	Practical significance for planners
Graph $F_{n}\times P_{2}$	Two-period conference model	Foundational structural framework
Central vertices	Coordinator sessions	Main scheduling constraint
Peripheral vertices	Team sessions	Team-specific resource requirement and task allocations
Horizontal edges	Concurrent session conflicts	Prevent concurrent room use for related sessions
Vertical edges	Cross-period session constraints	Assure no room is reused by the same team for sequential time slots
$P_{n}\left(k\right)$	Number of conflict-free schedules	Primary Flexibility Metric: Higher values indicate greater resilience to changes
χ=3	Absolute minimum room requirement	Feasibility Guarantee: Establishes a strict lower bound for resource allocation
ψ(k)	Flexibility multiplier per team	Scalability Predictor: Enables forecasting of resource needs as the conference grows
Real root of ψ(k) in [2,3]	Theoretical k threshold	Planning Threshold: Highlights the critical transition from infeasible ( k=2) to feasible (k=3) scheduling

The mapping elucidates the following core principles:

• The graph $F_{n}\times P_{2}$ serves as the foundational structural model for the entire two-period conference.
• Its vertices directly represent physical scheduling entities: central vertices correspond to coordinator sessions, while peripheral vertices represent team-specific sessions, thereby defining the core resource allocation requirements.
• The edges explicitly model the two critical types of scheduling conflicts: horizontal edges prevent concurrent room usage for related sessions, and vertical edges enforce the constraint that a team cannot reuse the same room across consecutive time periods.
• Most importantly, the key algebraic results—the chromatic polynomial $P_{n}\left(k\right)$ , the chromatic number χ=3 , the transition polynomial ψ(k) , and its real root in [2,3] —are transformed into practical planning tools. These tools quantitatively measure flexibility, guarantee minimum resource feasibility, predict scalability, and identify critical resource thresholds.

This structured translation equips planners with a clear, actionable methodology. It enables them to leverage the rigorous guarantees and predictive power of graph theory to make informed, concrete scheduling decisions and formulate robust, mathematically-grounded resource allocation strategies.

**4.5.7 Model Limitations and Assumptions**

1. All teams have identical scheduling constraints.
2. Meeting rooms are homogeneous and interchangeable.
3. No additional temporal constraints beyond the two-period framework.
4. The model assumes complete conflict graphs without probabilistic elements.

## 5. Discussion

### 5.1 Theoretical contribution

This work provides a complete algebraic characterization of the chromatic polynomial for $F_{n}\times P_{2}$ . The recurrence relation $P_{n}\left(k\right)=\psi \left(k\right)\;P_{n-1}\left(k\right)$ establishes ψ(k) as a new graph invariant that encodes the recursive constraint structure of this graph family. Crucially, the existence and constancy of this invariant stem from the structural symmetry and conditional independence of the recursively attached blocks $B_{n}$ —a property formalized in Lemma 4.1.4. Unlike well-documented results for standard Cartesian products, ^9, 12^ this addresses a previously unexplored structure. Furthermore, the stability of the chromatic number at χ=3 despite linear growth in order (|V|=4n+2 ), highlights how restrictive local features determine global properties—a fundamental decoupling of structural scale from chromatic complexity. ^6, 7^

### 5.2 Practical utility

The chromatic polynomial serves as a direct quantitative tool in our two-period conference scheduling model, building upon established graph-coloring paradigms. ^1, 13^ The closed-form expression $P_{n}\left(k\right)={\left[\psi \left(k\right)\right]}^{n-2}\;P_{2}\left(k\right)$ reveals ψ(k) as a flexibility multiplier: each additional room increases feasible schedules by a factor of ψ(k) per team. For instance, moving from k=3 to k=4 rooms yields a ψ(4)=22 -fold increase in valid allocations, as quantified in Table 4, transforming an abstract invariant into a practical decision-support metric.

From a computational perspective, the recurrence enables substantial efficiency gains. Computing $P_{n}\left(k\right)$ via exponentiation by squaring reduces the time complexity from exponential (under deletion–contraction) to O(logn) . This demonstrates how structural insights into graph families can yield algorithmic improvements, rendering an otherwise intractable problem practical for large n . In scheduling terms, evaluating $P_{100}\left(k\right)$ requires only about seven multiplications—feasible for real-time planning—whereas generic methods would be prohibitive.

Thus, the work bridges pure graph theory with applied computation: the same algebraic invariant that elucidates the chromatic structure also enables efficient enumeration, directly informing both algorithmic design and resource-allocation decisions in constrained environments.

### 5.3 Limitations and future directions

The model assumes identical resources and a two-period framework, suggesting natural extensions:

1. Generalization to $F_{n}\times P_{m}$ for m>2 for multi-period scheduling.
2. Enhanced models incorporating heterogeneous resources via list-chromatic polynomials or weighted coloring models.
3. Theoretical connections between the asymptotic distribution of chromatic roots and phase transitions in the Potts model. ^16^

## Ethical considerations

This study does not involve human participants, animal subjects, or sensitive data. Therefore, no ethical approval was required.

## Use of AI-assisted technology

During manuscript revision, DeepSeek (deepseek.com) was used as a supplementary tool for language editing and algebraic verification. The authors critically reviewed all output and take full responsibility for the final work.

## Data Availability

This is a theoretical study in algebraic graph theory. All results, including the recurrence relation, the closed-form expression for the chromatic polynomial, and all numerical values, are derived analytically and presented within the article. No external datasets were generated or analyzed. All findings are fully reproducible using the formulas and methods provided in Sections 3 and 4. This is a theoretical mathematical study and does not involve clinical trials, animal experiments, observational studies, or qualitative research. Therefore, no specific reporting guidelines (e.g., CONSORT, ARRIVE, STROBE, COREQ) are applicable.
