# Chromatic Polynomials of *F* *n*× *P*2  Graphs: Algebraic Analysis and Scheduling Applications

**DOI:** 10.12688/f1000research.176896.2

**Published:** 2026-06-12

**Authors:** Sarah M. Talab, Nabeel E. Arif

**Affiliations:** 1 Mathematics, Tikrit University, Tikrit, Saladin Governorate, 34001, Iraq

**Keywords:** Graph coloring, Chromatic polynomial, Cartesian product, Friendship graph, Combinatorial mathematics, Recurrence relation, Closed-form expression, Scheduling.

## Abstract

**Background:**

Chromatic polynomials are fundamental algebraic invariants in graph theory, linking structural properties of graphs with algebraic and enumerative information. While extensive results exist for paths, cycles, and several classical graph products, the Cartesian product $F_{n}\times P_{2}$ , where $F_{n}$ is the friendship graph, and $P_{2}$ is the path on two vertices, has received limited direct attention despite its layered triangular structure.

**Methods:**

We use a recursive block decomposition of $F_{n}\times P_{2}$ . After fixing the colors of the two central vertices, we derive the local extension count for the newly attached block through a structured combinatorial case analysis. This yields the transition polynomial ψ(k) , which governs both the recurrence relation and the closed-form expression for the chromatic polynomial.

**Results:**

We establish the recurrence relation $P_{n}\left(k\right)=\psi \left(k\right)P_{n-1}\left(k\right)$ and the closed-form expression $P_{n}\left(k\right)={\left[\psi \left(k\right)\right]}^{n-2}P_{2}\left(k\right),$ where $\psi \left(k\right)=k^{4}-8k^{3}+26k^{2}-41k+26.$ We prove that the chromatic number is $\chi \left(F_{n}\times P_{2}\right)=3.$ The transition polynomial ψ(k) has exactly two real roots, both lying in the interval [2,3] . The asymptotic behavior is given by $\lim_{n\to \infty }|P_{n}\left(k\right){|}^{\frac{1}{n}}=|\psi \left(k\right)|$ for fixed k with $P_{2}\left(k\right)\neq 0$ . Numerical computations for selected integer values of k confirm the recurrence and closed-form formula.

**Conclusions:**

This work provides an algebraic characterization of the chromatic polynomial of $F_{n}\times P_{2}$ through a recursive block structure and a single transition polynomial. The results also support an illustrative two-period scheduling interpretation, where the chromatic polynomial counts feasible room assignments under explicitly stated conflict constraints.

## 1. Introduction

Chromatic polynomials are fundamental objects in algebraic graph theory. Introduced by Birkhoff (1912) in connection with the four-color problem, they were further developed through Whitney’s (1932) deletion–contraction recurrence and Read’s (1968) systematic studies, culminating in the comprehensive treatment of Tutte and Read (1988). For a graph G , the chromatic polynomial P(G,k) counts the number of proper k -colorings of G , thereby connecting combinatorial structure with algebraic and enumerative properties.

Beyond their theoretical importance, chromatic polynomials and graph coloring methods arise in several applied settings, including task scheduling, ^1^ data analysis, ^2^ network design, ^3^ theoretical chemistry, ^4^ and statistical physics. ^5^ However, computing the chromatic polynomial is #P-hard in general. This difficulty is especially relevant for graph Cartesian products, where the chromatic polynomial of a product graph is not determined by a simple formula involving only the chromatic polynomials of its factors. Consequently, explicit formulas are usually obtained only for structured graph families.

Cartesian products of graphs provide an important setting for studying layered and composite structures. Their structural and coloring properties have been investigated extensively. ^6^ ^,^ ^7^ Recent studies have derived chromatic polynomials for particular families such as triangular snake graphs, n -centipede graphs, layered graphs, and grid-type products using recursive, transfer-matrix, or decomposition-based methods. ^8^ ^–^ ^12^

While many results are known for Cartesian products involving paths and cycles, the graph family $F_{n}\times P_{2}$ , formed from the friendship graph $F_{n}$ and the path $P_{2}$ , has received limited direct attention. This family combines local triangular clusters with a two-layer product structure, making it suitable for recursive chromatic analysis.

This paper provides an analytical framework for $F_{n}\times P_{2}$ by:

1. deriving a recurrence relation and closed-form expression for its chromatic polynomial;
2. establishing its structural properties and chromatic number;
3. analyzing the transition polynomial, its real roots, and the asymptotic growth rate;
4. validating the formulas through numerical computation;
5. presenting an illustrative two-period scheduling interpretation based on the graph-coloring model.

The scheduling interpretation is motivated by established graph-coloring approaches to scheduling and resource allocation 1, 13, and illustrates how the derived chromatic polynomial can be used to quantify feasible room assignments in a two-period resource-allocation setting with explicitly defined conflict constraints. This applied perspective complements the algebraic results by showing how the recurrence and closed-form expression translate into concrete scheduling counts.

## 2. Preliminaries

Definition 2.1 ^14^: The friendship graph $F_{n}$ , for n≥2 , is defined as the union of n copies of $C_{3}$ sharing a common vertex. This common vertex is called the central vertex.

Formally:

$$V\left(F_{n}\right)=\left\{v_{0}\right\}\cup {\left\{u_{i},w_{i}\right\}}_{i=1}^{n}$$

where $v_{0}$ is the center vertex, and

$$E\left(F_{n}\right)={\left\{(v_{0},u_{i}),(v_{0},w_{i}),(u_{i},w_{i})\right\}}_{i=1}^{n}.$$

Hence,

$$|V\left(F_{n}\right)|=2n+1,$$

and

$$|E\left(F_{n}\right)|=3n.$$

Figure 1 (Friendship graphs *F*_*n*_):

Definition 2.2 ^6^: The Cartesian product of two graphs G and H , denoted by G×H , is the graph whose vertex set is V(G)×V(H) , with two vertices $\left(u_{1},w_{1}\right)$ and $\left(u_{2},w_{2}\right)$ adjacent if:

•
$u_{1}=u_{2}\;$ and $w_{1}$ is adjacent to $w_{2}$ in H , or
•
$w_{1}=w_{2}$ and $u_{1}$ is adjacent to $u_{2}$ in G .

Throughout this paper, the symbol × denotes the Cartesian product of graphs.

Definition 2.3 ^15^: The chromatic polynomial P(G,k) is a polynomial in k that expresses the number of proper vertex k -colorings of G , such that adjacent vertices share distinct colors.

Definition 2.4: The graph $F_{n}\times P_{2}$ is the Cartesian product of a friendship graph $\;F_{n}$ with a path $P_{2}$ , forming two parallel layers of $\;F_{n}$ with corresponding vertices connected by edges.

Figure 2 (Cartesian product *F*_*n*_ × *P*_2_):

**Figure 1.**
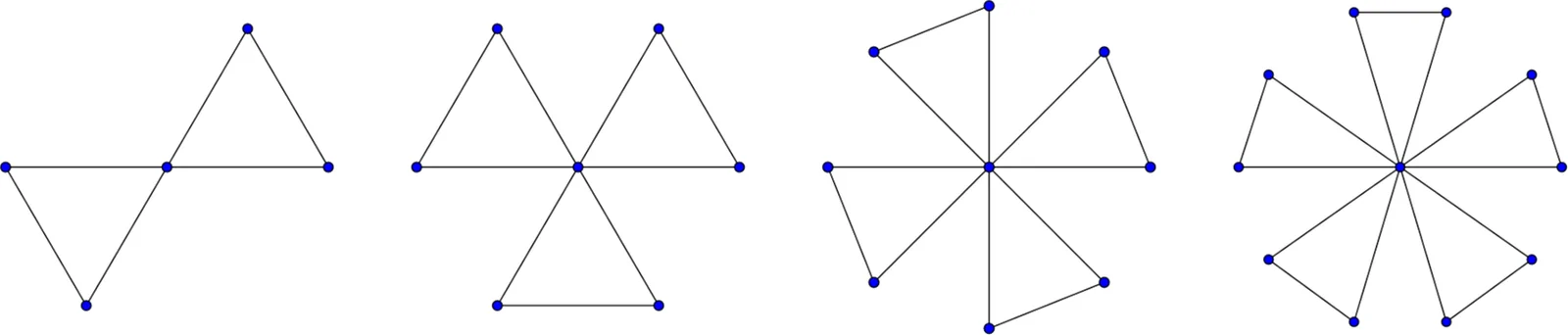
Friendship graphs $F_{n}$ for n=2,3,4,5. Each graph is formed by n triangles sharing a common central vertex $v_{0}$ , illustrating the recursive structure of the friendship graph family.

**Figure 2.**
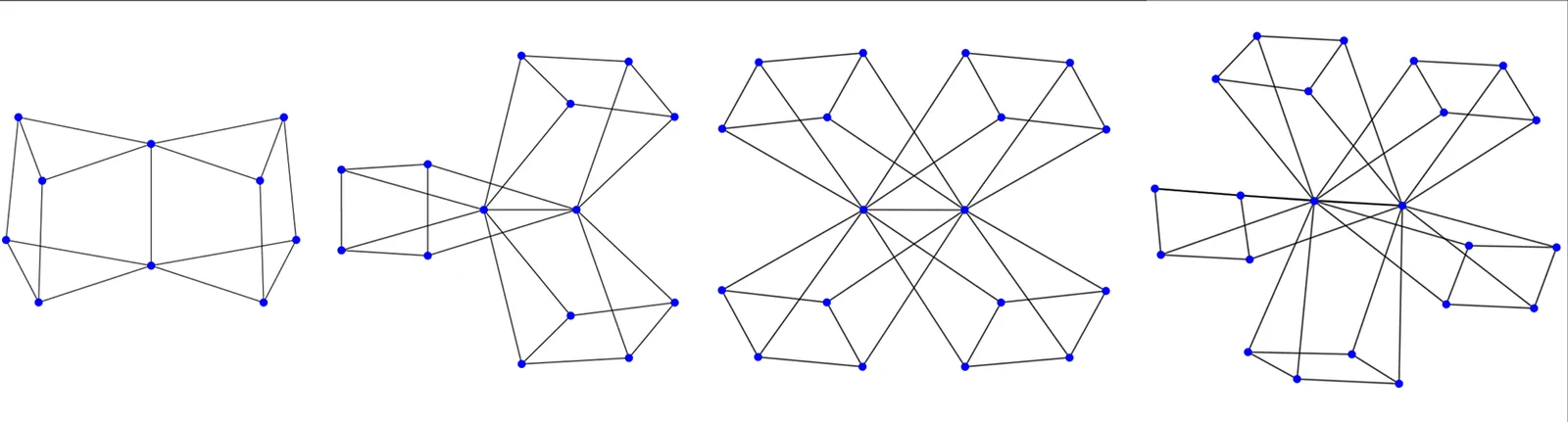
Structure of the Cartesian Product $F_{n}\times P_{2}$ for n=2,3,4,5. This construction yields two parallel layers of $F_{n}$ , with corresponding vertices connected by vertical edges.

## 3. Methods

### 3.1 Analytical framework and structural decomposition

This is a theoretical study in algebraic and combinatorial graph theory, analyzing the chromatic polynomial of the graph family $G_{n}=F_{n}\times P_{2}.$ The core of our approach is a structural decomposition that reveals a recursive block construction. The graph $G_{n}$ consists of two copies of $F_{n}$ , denoted by layers A and B , with corresponding vertices connected by vertical edges. For n≥3 , the graph $G_{n}$ is obtained from $G_{n-1}$ by attaching a new block $B_{n}$ . This block contains the four new peripheral vertices $\left\{u_{n}^{A},w_{n}^{A},u_{n}^{B},w_{n}^{B}\right\}$ together with the edges forming the two new triangles, one in each layer, and the two vertical edges between corresponding peripheral vertices. The block $B_{n}$ meets the previously constructed graph only through the two central vertices $v_{0}^{A}$ and $v_{0}^{B}$ . This localized attachment is the key structural feature that isolates the chromatic contribution of each recursive step.

Lemma 3.1.1 (Conditional independence of the recursive block)

Let $B_{n}$ be the block added when passing from $G_{n-1}$ to $G_{n}$ in the recursive construction of $G_{n}=F_{n}\times P_{2}.$ The block $B_{n}$ contains the four new peripheral vertices $\left\{u_{n}^{A},w_{n}^{A},u_{n}^{B},w_{n}^{B}\right\}.$ The only vertices of $G_{n-1}$ adjacent to vertices of $B_{n}$ are the two central vertices $v_{0}^{A},\;v_{0}^{B}.$ Consequently, once distinct colors are assigned to $v_{0}^{A}$ and $v_{0}^{B}$ , every coloring constraint involving the new peripheral vertices is local to $B_{n}$ . Hence the number of proper extensions to $B_{n}$ depends only on the number of colors k , and is denoted by

$$N_{\text{diff}}\left(k\right).$$

**Proof:** By construction, the n -th triangle of the friendship graph shares only the central vertex $v_{0}$ with the preceding triangles. Therefore, in the Cartesian product $F_{n}\times P_{2}$ , no new peripheral vertex in $B_{n}$ is adjacent to any peripheral vertex of an earlier block.

The only connections between the new block and the previously constructed graph occur through the two central vertices $v_{0}^{A},v_{0}^{B}.$ Thus, after the colors of $v_{0}^{A}$ and $v_{0}^{B}$ are fixed, the coloring constraints involving the four new vertices are exactly the triangle constraints in the two layers together with the two vertical constraints between corresponding new peripheral vertices. None of these constraints involves a peripheral vertex from any earlier block.

Since $v_{0}^{A}$ and $v_{0}^{B}$ are adjacent, they receive distinct colors in every proper coloring. Moreover, by symmetry of the color palette, the number of valid completions depends only on k , not on the particular labels assigned to $v_{0}^{A}$ and $v_{0}^{B}$ . Therefore, every proper coloring of $G_{n-1}$ admits the same number of extensions to $B_{n}$ , namely $N_{\text{diff}}\left(k\right)$ . This proves the claim. ∎

### 3.2. Combinatorial derivation of the transition polynomial ψ(k)

We now compute $N_{\text{diff}}\left(k\right)$ , the number of admissible color extensions to the block $B_{n}$ . Since $v_{0}^{A}$ and $v_{0}^{B}$ are adjacent, we may assume by symmetry that

$$c\left(v_{0}^{A}\right)=1,\;c\left(v_{0}^{B}\right)=2.$$

Put

$$x=c\left(u_{n}^{A}\right),\;y=c\left(w_{n}^{A}\right),\;z=c\left(u_{n}^{B}\right),\;t=c\left(w_{n}^{B}\right).$$

The coloring constraints are

x≠1,y≠1,x≠y,

z≠2,t≠2,z≠t,

and

x≠z,y≠t.

Thus $N_{\text{diff}}\left(k\right)$ is the number of ordered quadruples (x,y,z,t) satisfying these constraints. We count according to whether the ordered pair (x,y) contains the color 2 .

**Case 1. The pair** (x,y) **contains the color** 2

Since x≠y , the color 2 appears in exactly one position. The other color is chosen from the k−2 colors different from 1 and 2 . Hence there are

2(k−2)

choices for ( *x*, *y*).

For each such choice, the number of admissible choices for (z,t) is ${\left(k-2\right)}^{2}$ . Indeed, if x=2 and y=r , where r∉{1,2} , then z≠2 , while t≠2,r . Before imposing z≠t , this gives

(k−1)(k−2)

choices. The forbidden equality z=t.  occurs for exactly k−2 common colors, namely all colors different from

2

and

r

Therefore,

$$\left(k-1\right)\left(k-2\right)-\left(k-2\right)={\left(k-2\right)}^{2}.$$

The same count holds when y=2 . Thus, the contribution of this case is $\;2{\left(k-2\right)}^{3}.$

**Case 2. The pair** (x,y) **does not contain the color** 2

In this case, *x* and *y* are distinct colors chosen from the *k*−2 colors different from 1 and 2

Hence

(k−2)(k−3)

choices for ( *x*, *y*).

For fixed such x and y , the color z must avoid 2 and x , while t must avoid 2 and y . Hence, before imposing z≠t , there are ${\left(k-2\right)}^{2}$

choices for ( *z*, *t*). The forbidden equality *z* = *t* occurs when the common color is different from 2, x, and y, giving k − 3 forbidden choices. Therefore, the number of valid choices for (z, t) is

$${\left(k-2\right)}^{2}-\left(k-3\right)=k^{2}-5k+7.$$

Thus, the contribution of this case is

$$\left(k-2\right)\left(k-3\right)\left(k^{2}-5k+7\right).$$

Combining the two cases, we obtain

$$N_{\text{diff}}\left(k\right)=2{\left(k-2\right)}^{3}+\left(k-2\right)\left(k-3\right)\left(k^{2}-5k+7\right).$$

Expanding gives

$$\begin{array}{cc} N_{\text{diff}}\left(k\right) & =2{\left(k-2\right)}^{3}+\left(k-2\right)\left(k-3\right)\left(k^{2}-5k+7\right)\\ & \;=k^{4}-8k^{3}+26k^{2}-41k+26. \end{array}$$

Therefore

$$N_{\text{diff}}\left(k\right)=\psi \left(k\right),$$

where

$$\psi \left(k\right)=k^{4}-8k^{3}+26k^{2}-41k+26.$$

The enumeration is summarized in Table 1.

**Table 1.** Case analysis for the local extension count $N_{\text{diff}}\left(k\right)=\psi \left(k\right)$ .

Case	Choices for (x,y)	Choices for (z,t)	Contribution
(x,y) contains color 2	2(k−2)	${\left(k-2\right)}^{2}$	$2{\left(k-2\right)}^{3}$
(x,y) does not contain color 2	(k−2)(k−3)	$k^{2}-5k+7$	$\left(k-2)\left(k-3\right)(k^{2}-5k+7\right)$

As an independent algebraic verification, direct symbolic computation gives

$$P_{3}\left(k\right)=\psi \left(k\right)P_{2}\left(k\right),$$

which confirms the consistency of this direct combinatorial derivation.

### 3.3. Derivation of the recurrence and closed-form expression

Let $P_{n}\left(k\right)$ denote the chromatic polynomial of $G_{n}$ . By Lemma 3.1.1, every proper k -coloring of $G_{n-1}$ can be extended to the new block in exactly $N_{\text{diff}}\left(k\right)$ ways. From the direct enumeration in Section 3.2 ,

$$N_{\text{diff}}\left(k\right)=\psi \left(k\right),$$

where

$$\psi \left(k\right)=k^{4}-8k^{3}+26k^{2}-41k+26.$$

Hence, for n≥3

$$P_{n}\left(k\right)=N_{\text{diff}}\left(k\right)P_{n-1}\left(k\right)=\psi \left(k\right)P_{n-1}\left(k\right).$$

Since both sides are polynomials in k and the equality holds for all positive integers k , the recurrence holds as a polynomial identity.

Applying this recurrence repeatedly gives

$$P_{n}\left(k\right)={\left[\psi \left(k\right)\right]}^{n-2}P_{2}\left(k\right),\;n\geq 2.$$

This proves both the first-order recurrence and the closed-form expression for $F_{n}\times P_{2}$ .

## 4. Results

### 4.1 Structural analysis of $F_{n}\times P_{2}$

Theorem 4.1.1: (Structural Properties of $F_{n}\times P_{2}$ )

For n≥2 , the graph $F_{n}\times P_{2}$ possesses the following structural properties, which directly affect its chromatic behavior:

1.
|V|=4n+2.

2.
|E|=8n+1 .
3. Degree sequence $\left\{{\left(2n+1\right)}^{\left(2\right)},\;3^{\left(4n\right)}\right\}$ .

Where the notation $\;d^{\left(\eta \right)}$ denotes (η) vertices of degree d .

Proof:

1. **Vertex count**: The friendship graph $F_{n}$ has (2n+1) vertices. Since $P_{2}$ has two vertices, the Cartesian product has
$$|V\left(F_{n}\times P_{2}\right)|=2\left(2n+1\right)=4n+2.$$
2. **Edge count**: The graph
  $$F_{n}\times P_{2},$$
  consists of two copies of
  $$F_{n}$$,
  giving
$$2|E\left(F_{n}\right)|=2\left(3n\right)=6n$$
  internal edges. In addition, there is one vertical edge joining the two copies of each vertex of
  $$F_{n}$$,
  giving
$$|V\left(F_{n}\right)|=2n+1$$
  vertical edges. Therefore,
$$|E\left(F_{n}\times P_{2}\right)|=6n+\left(2n+1\right)=8n+1.$$
3. **Connectivity**: Since $F_{n}$ is connected and $P_{2}$ is connected, their Cartesian product $F_{n}\times P_{2}$ is connected. Therefore, $F_{n}\times P_{2}$ is connected for all n≥2 .

4. **Degree sequence:** There are two central vertices, one in each layer. Each central vertex is adjacent to 2n peripheral vertices in its own layer and to the corresponding central vertex in the other layer. Hence each central vertex has degree

2n+1.

There are 4n peripheral vertices in total. Each peripheral vertex is adjacent to one central vertex in its layer, one partner vertex in the same triangle, and its corresponding vertex in the other layer. Hence each peripheral vertex has degree 3.

Therefore, the degree sequence is

$$\;\left\{{\left(2n+1\right)}^{\left(2\right)},3^{\left(4n\right)}\right\}.\;∎$$

Remark 4.1.2: (Edge classification in $F_{n}\times P_{2}$ )

The edge count in Theorem 4.1.1 can be refined by classifying the edges of $F_{n}\times P_{2}$ into three types, as shown in Table 2. There is one central edge joining the two central vertices. The center-peripheral edges consist of the 2n edges incident with the center in each layer, giving 4n edges in total. The remaining peripheral edges consist of the 2n triangle-base edges in the two layers and the 2n vertical edges between corresponding peripheral vertices, giving 4n edges. Hence,

**Table 2.** Edge classification in $F_{n}\times P_{2}$ .

Edge type	Description	Degree of endpoints	Count
Central edge	Connects the two central vertices $v_{0}^{A}$ and $v_{0}^{B}$	(2n+1,2n+1)	1
Center-peripheral edges	Connect central vertices to peripheral vertices within each layer	(2n+1,3)	4n
Peripheral-peripheral edges	Include triangle-base edges and peripheral vertical edges	(3,3)	4n

1+4n+4n=8n+1,

which agrees with the edge count in Theorem 4.1.1.

### 4.2 Chromatic polynomial analysis

Theorem 4.2.1: (Recurrence Relation)

For all n≥3 , the chromatic polynomial of $F_{n}\times P_{2}$ satisfies a first-order linear recurrence

$$P_{n}\left(k\right)=\psi \left(k\right)\;P_{n-1}\left(k\right),$$

where

$$\psi \left(k\right)=k^{4}-8k^{3}+26k^{2}-41k+26.$$

Proof: By Lemma 3.1.1, once the colors of the two central vertices are fixed, the number of admissible extensions to the newly attached block $B_{n}$ is independent of the coloring of $G_{n-1}$ . Hence,

$$P_{n}\left(k\right)=N_{\text{diff}}\left(k\right)P_{n-1}\left(k\right).$$

By the direct combinatorial enumeration in Section 3.2,

$$N_{\text{diff}}\left(k\right)=\psi \left(k\right).$$

Therefore,

$$P_{n}\left(k\right)=\psi \left(k\right)P_{n-1}\left(k\right),$$

as required. ∎

Theorem 4.2.2: (Closed-Form Expression)

For n≥2 , the chromatic polynomial of $F_{n}\times P_{2}$ is given by

$$P_{n}\left(k\right)={\left[\psi \left(k\right)\right]}^{n-2}\;P_{2}\left(k\right).$$

Proof: We prove the formula by induction on n . For n=2 , we have

$$P_{2}\left(k\right)={\left[\psi \left(k\right)\right]}^{0}P_{2}\left(k\right),$$

so, the formula holds.

Assume that the formula holds for n−1 , where n≥3 . That is,

$$P_{n-1}\left(k\right)={\left[\psi \left(k\right)\right]}^{n-3}P_{2}\left(k\right).$$

Using the recurrence relation from Theorem 4.2.1,

$$P_{n}\left(k\right)=\psi \left(k\right)P_{n-1}\left(k\right),$$

we obtain

$$P_{n}\left(k\right)=\psi \left(k\right){\left[\psi \left(k\right)\right]}^{n-3}P_{2}\left(k\right)={\left[\psi \left(k\right)\right]}^{n-2}P_{2}\left(k\right).$$

Therefore, the formula holds for all n≥2 . ∎

Corollary 4.2.3 (Computational Efficiency for Fixed k )

For a fixed numerical value of k , the closed-form expression

$$P_{n}\left(k\right)={\left[\psi \left(k\right)\right]}^{n-2}P_{2}\left(k\right)$$

allows $P_{n}\left(k\right)$ to be evaluated using O(logn) arithmetic multiplications by applying exponentiation by squaring to ${\left[\psi \left(k\right)\right]}^{n-2}$ .

This complexity statement concerns numerical evaluation at a fixed value of *k*. It should not be interpreted as a bound for computing the fully expanded symbolic polynomial $P_{n}\left(k\right)$ , since the degree and output size of the polynomial increase with

n

Indeed,

$$\deg\;P_{n}\left(k\right)=\;\deg\;P_{2}\left(k\right)+\left(n-2\right)\;\deg\;\psi \left(k\right).$$

Since $\;\deg\;P_{2}\left(k\right)=10$ and degψ(k)=4 , we obtain

$$\deg\;P_{n}\left(k\right)=10+4\left(n-2\right)=4n+2.$$

Thus, the closed-form expression gives an efficient method for fixed- k numerical evaluation, whereas the fully expanded symbolic polynomial has size growing with n .

Proposition 4.2.4 (Chromatic Number)

For all n≥2 ,

$$\chi \left(F_{n}\times P_{2}\right)=3$$

Proof: Since $F_{n}\times P_{2}$ contains copies of $C_{3}$ , at least three colors are required. Hence,

$$\chi \left(F_{n}\times P_{2}\right)\geq 3.$$

It remains to show that three colors are sufficient. Define a coloring $c:V\left(F_{n}\times P_{2}\right)\to \left\{1,2,3\right\}$ as follows:

$$c\left(v_{0}^{A}\right)=1,\;c\left(v_{0}^{B}\right)=2.$$

For each i=1,…,n , assign

$$c\left(u_{i}^{A}\right)=2,\;c\left(w_{i}^{A}\right)=3,$$

and

$$c\left(u_{i}^{B}\right)=3,\;c\left(w_{i}^{B}\right)=1.$$

In layer A , each triangle $\left(v_{0}^{A},u_{i}^{A},w_{i}^{A}\right)$ receives the three distinct colors 1,2,3 . In layer B , each triangle $\left(v_{0}^{B},u_{i}^{B},w_{i}^{B}\right)$ receives the three distinct colors 2,3,1 . The central vertical edge satisfies

$$c\left(v_{0}^{A}\right)\neq c\left(v_{0}^{B}\right),$$

and the peripheral vertical edges satisfy

$$c\left(u_{i}^{A}\right)\neq c\left(u_{i}^{B}\right),\;c\left(w_{i}^{A}\right)\neq c\left(w_{i}^{B}\right)$$

for every i . Therefore, all adjacent vertices receive distinct colors, so c is a proper 3-coloring. Hence,

$$\chi \left(F_{n}\times P_{2}\right)\leq 3.$$

Combining the lower and upper bounds gives

$$\;\chi \left(F_{n}\times P_{2}\right)=3.\;∎$$

### 4.3 Algebraic and asymptotic analysis

Theorem 4.3.1 (Real Roots of ψ(k))

The chromatic transition polynomial

$$\psi \left(k\right)=k^{4}-8k^{3}+26k^{2}-41k+26$$

has exactly two real roots. One root is k=2 , and the other lies in the interval (2,2.5) . In particular, both real roots lie in [2,3] .

Proof: Direct evaluation gives

ψ(2)=0 , ψ(2.5)=0.0625>0 , ψ(3)=2>0 .

The first and second derivatives are

$$\psi^{\prime }\left(k\right)=4k^{3}-24k^{2}+52k-41,$$

and

$$\psi^{\prime \prime }\left(k\right)=12k^{2}-48k+52.$$

The discriminant of $\psi^{\prime \prime }\left(k\right)$ is

$${\left(-48\right)}^{2}-4\left(12\right)\left(52\right)=-192<0.$$

Since the leading coefficient of $\psi^{\prime \prime }\left(k\right)$ is positive, it follows that

$$\psi^{\prime \prime }\left(k\right)>0$$

for all real k . Hence $\psi^{\prime }\left(k\right)$ is strictly increasing on ℝ , and ψ(k) is strictly convex.

Moreover,

$$\psi^{\prime }\left(2\right)=-1<0,\;\psi^{\prime }\left(2.5\right)=1.5>0.$$

Therefore, $\psi^{\prime }\left(k\right)$ has a unique zero α∈(2,2.5) . Thus, ψ(k) is strictly decreasing on (−∞,α) and strictly increasing on (α,∞) .

Since ψ(2)=0 and $\psi^{\prime }\left(2\right)<0$ , the polynomial becomes negative immediately to the right of 2 . On the other hand, ψ(2.5)>0 . Hence, by the Intermediate Value Theorem, ψ(k) has a root ρ∈(2,2.5) .

Finally, because ψ(k) is decreasing before α and increasing after α , it can have at most one real root on each side of α . Since k=2 is one root and ρ∈(2,2.5) is another, these are the only real roots of ψ(k) . The remaining two roots are non-real complex conjugates. ∎

Theorem 4.3.2 (Exponential Growth Rate)

Let

k∈ℂ

be fixed and suppose that

$$P_{2}\left(k\right)\neq 0$$.

Then

$$\lim_{n\to \infty }|P_{n}\left(k\right){|}^{\frac{1}{n}}=|\psi \left(k\right)|.$$

In particular, for every integer k≥3 ,

$$\lim_{n\to \infty }P_{n}{\left(k\right)}^{\frac{1}{n}}=\psi \left(k\right).$$

Proof: From the closed-form expression in Theorem 4.2.2, we have

$$P_{n}\left(k\right)={\left[\psi \left(k\right)\right]}^{n-2}\;P_{2}\left(k\right)\;\left(n\;\geq \;2\right).$$

Taking absolute values gives

$$|P_{n}\left(k\right)|=|\psi \left(k\right){|}^{n-2}|P_{2}\left(k\right)|.$$

Hence,

$$|P_{n}\left(k\right){|}^{\frac{1}{n}}=|\psi \left(k\right){|}^{\frac{\left(n-2\right)}{n}}|P_{2}\left(k\right){|}^{\frac{1}{n}}.$$

Since $\frac{n-2}{n}\to 1$ and, because $P_{2}\left(k\right)\neq 0$ ,

$$|P_{2}\left(k\right){|}^{\frac{1}{n}}\to 1,$$

we obtain

$$\lim_{n\to \infty }|P_{n}\left(k\right){|}^{\frac{1}{n}}=|\psi \left(k\right)|.$$

For integer

k≥3,

$$P_{n}\left(k\right)$$

counts proper

k

-colorings, so

$$P_{n}\left(k\right)>0$$.

Also,

$$\psi \left(k\right)=N_{\text{diff}}\left(k\right)>0$$,

since it counts the number of admissible extensions to one additional block. Therefore

$$|P_{n}\left(k\right)|=P_{n}\left(k\right),\;|\psi \left(k\right)|=\psi \left(k\right),$$

and consequently

$$\lim_{n\to \infty }P_{n}{\left(k\right)}^{\frac{1}{n}}=\psi \left(k\right).$$

This proves the result. ∎

Remark 4.3.3 (Absolute Values and the Combinatorial Range)

The absolute values are necessary when k is treated as a real or complex parameter, since $P_{n}\left(k\right)$ may be negative or complex-valued. The simpler expression

$$\lim_{n\to \infty }P_{n}{\left(k\right)}^{\frac{1}{n}}=\psi \left(k\right).$$

is valid in the combinatorial range k∈ℤ , k≥3 , where both $P_{n}\left(k\right)$ and ψ(k) are positive.

Corollary 4.3.4 (Ratio Convergence of Chromatic Polynomials)

For every fixed k such that ψ(k)≠0 and $P_{2}\left(k\right)\neq 0$ ,

$$\lim_{n\to \infty }\frac{P_{n}\left(k\right)}{P_{n-1}\left(k\right)}=\psi \left(k\right).$$

Proof: By Theorem 4.2.1,

$$P_{n}\left(k\right)=\psi \left(k\right)P_{n-1}\left(k\right),\;n\geq 3.$$

Since

ψ(k)≠0

and

$$P_{2}\left(k\right)\neq 0$$,

the closed-form expression implies

$$P_{n-1}\left(k\right)\neq 0$$

for all

n≥3

Hence

$$\frac{P_{n}\left(k\right)}{P_{n-1}\left(k\right)}=\psi \left(k\right),$$

and the stated limit follows immediately. ∎

**Interpretation**

The value |ψ(k)| is the exponential growth constant of the sequence $\left\{P_{n}\left(k\right)\right\}$ for fixed k with $P_{2}\left(k\right)\neq 0$ . In the combinatorial range k∈ℤ , k≥3 , the recurrence gives the exact relation

$$P_{n}\left(k\right)=\psi \left(k\right)P_{n-1}\left(k\right),$$

so, each additional block multiplies the number of proper k -colorings by ψ(k) .

Remark 4.3.5 (Root distribution)

From the closed-form expression in Theorem 4.2.2, we have

$$P_{n}\left(k\right)={\left[\psi \left(k\right)\right]}^{n-2}P_{2}\left(k\right).$$

Therefore, the zero set of

$$P_{n}\left(k\right)$$

is contained in the union of the zero set of

$$P_{2}\left(k\right)$$

and the zero set of

ψ(k).

More precisely, if

r

is a root of

ψ(k)

of multiplicity

$$m_{\psi }\left(r\right)$$

and a root of

$$P_{2}\left(k\right)$$

of multiplicity

$$m_{2}\left(r\right)$$,

then its multiplicity as a root of

$$P_{n}\left(k\right)$$

is

$$m_{2}\left(r\right)+\left(n-2\right)m_{\psi }\left(r\right).$$

If r is a root of ψ(k) but not a root of $P_{2}\left(k\right)$ , then its multiplicity in $P_{n}\left(k\right)$ is $\left(n-2\right)m_{\psi }\left(r\right)$ . Hence, as n increases, the locations of the roots remain in a fixed finite set, while the multiplicities of the roots contributed by ψ(k) grow linearly with n .

### 4.4 Numerical validation

To verify the recurrence relation $P_{n}\left(k\right)=\psi \left(k\right)P_{n-1}\left(k\right),$ from Theorem 4.2.1 and the closed-form expression $P_{n}\left(k\right)={\left[\psi \left(k\right)\right]}^{n-2}P_{2}\left(k\right)$ from Theorem 4.2.2, we computed the chromatic polynomials $P_{n}\left(k\right)$ for n=2,3,4,5 using **Wolfram Mathematica**.

For the tested integer values k=3,4,5,6 , the computations showed agreement among the following three approaches:

• direct computation of $P_{n}\left(k\right)$ ;
• evaluation using the recurrence relation;
• evaluation using the closed-form expression.

The numerical values of the transition polynomial ψ(k) and the chromatic polynomials $P_{2}\left(k\right)$ , $P_{3}\left(k\right)$ , $P_{4}\left(k\right)$ , and $P_{5}\left(k\right)$ are listed in Table 3.

**Table 3.** Numerical values of $P_{n}\left(k\right)$ for n=2,3,4,and5 , along with ψ(k) at integer values k≥3 .

k	ψ(k)	$P_{2}\left(k\right)$	$P_{3}\left(k\right)$	$P_{4}\left(k\right)$	$P_{5}\left(k\right)$
3	2	24	48	96	192
4	22	5,808	127,776	2,811,072	61,843,584
5	96	184,320	17,694,720	1,698,693,120	163,074,539,520
6	284	2,419,680	687,189,120	195,161,710,080	55,425,925,662,720

The following sample calculations illustrate the agreement:

• Theorem 4.2.1: $P_{3}\left(3\right)=\psi \left(3\right)P_{2}\left(3\right)=2\cdot 24=48.$

• Theorem 4.2.2: $P_{5}\left(6\right)={\left[\psi \left(6\right)\right]}^{3}P_{2}\left(6\right)=284^{3}\cdot 2,419,680=55,425,925,662,720.$

Moreover, the recurrence gives the exact growth ratio for fixed k :

$$\frac{P_{5}\left(4\right)}{P_{4}\left(4\right)}=\frac{61,843,584}{2,811,072}=22=\psi \left(4\right).$$

This exact ratio should be distinguished from the asymptotic n -th root behavior established in Theorem 4.3.2. In the positive integer coloring range k≥3 , that theorem gives

$$\lim_{n\to \infty }P_{n}{\left(k\right)}^{\frac{1}{n}}=\psi \left(k\right).$$

For finite n , however, the quantity $P_{n}{\left(k\right)}^{\frac{1}{n}}$ need not be close to ψ(k) . For example,

$$P_{5}{\left(4\right)}^{\frac{1}{5}}={\left(61,843,584\right)}^{\frac{1}{5}}\;\approx \;36.16,$$

whereas

ψ(4)=22.

The explicit polynomial expressions used as the basis for these computations are:

$$P_{2}\left(k\right)=k^{10}-17k^{9}+132k^{8}-614k^{7}+1882k^{6}-3932k^{5}+5581k^{4}-5165k^{3}+2808k^{2}-676k.$$

$$P_{3}\left(k\right)=k^{14}-25k^{13}+294k^{12}-2153k^{11}+10949k^{10}-40806k^{9}+114575k^{8}-245171k^{7}+399378k^{6}-488483k^{5}+435287k^{4}-266994k^{3}+100724k^{2}-17576k.$$

These computations provide computational confirmation of Theorem 4.2.1 and Theorem Theorem 4.2.2 for the tested values. They also illustrate the ratio identity $\frac{P_{n}\left(k\right)}{P_{n-1}\left(k\right)}=\psi \left(k\right)$ , which is an exact consequence of the recurrence for n≥3 . The asymptotic growth statement remains the limit result established in Theorem 4.3.2.

### 4.5 Illustrative application: a two-period scheduling model

This section presents a two-period scheduling interpretation in which the derived chromatic polynomial counts feasible room assignments under explicitly stated conflict constraints. The model is formulated under homogeneous-room and pairwise-conflict assumptions, providing a controlled resource-allocation setting for interpreting the recurrence relation and the closed-form expression.

**4.5.1 Model specification**

Consider a conference with one coordinator and n independent teams. The schedule is divided into two consecutive periods, denoted by A and B . The available resources are k identical meeting rooms.

The graph $G_{n}=F_{n}\times P_{2}$ is used as a conflict graph. Each vertex represents one session requiring a room, and each edge represents a pair of sessions that cannot be assigned to the same room. Thus, a proper k -coloring of $G_{n}$ corresponds to a valid assignment of k rooms to all sessions.

**4.5.2 Precise vertex-to-session mapping**

The vertex set of $G_{n}$ is

$$V\left(G_{n}\right)=\left\{v_{0}^{A},v_{0}^{B}\right\}\cup \left\{u_{i}^{A},w_{i}^{A},u_{i}^{B},w_{i}^{B}:\;1\;\leq \;i\;\leq \;n\right\}.$$

The two central vertices

$$v_{0}^{A},\;v_{0}^{B}$$

represent the coordinator sessions in periods A and B , respectively.

For each team i , the vertices

$$u_{i}^{A},\;w_{i}^{A}$$

represent two team-related sessions in period A , while

$$u_{i}^{B},\;w_{i}^{B}$$

represent the corresponding two team-related sessions in period B . Therefore, the total number of sessions is

2+4n=4n+2,

which agrees exactly with the vertex count of $F_{n}\times P_{2}$ .

**4.5.3 Edge-to-conflict mapping**

The edge set of $G_{n}$ encodes the scheduling constraints as follows.

For each i=1,…,n and each period X∈{A,B} , the edges

$$v_{0}^{X}u_{i}^{X},\;v_{0}^{X}w_{i}^{X}$$

represent coordinator-team conflicts within the same period. These sessions cannot be assigned to the same room.

The edge

$$u_{i}^{X}w_{i}^{X}$$

represents a conflict between the two team-related sessions of team i in the same period.

The vertical edges

$$v_{0}^{A}v_{0}^{B},\;u_{i}^{A}u_{i}^{B},\;w_{i}^{A}w_{i}^{B}$$

encode cross-period constraints between corresponding sessions. Under this modeling assumption, such paired sessions must receive distinct room assignments.

Thus, feasible schedules are exactly the proper

k

-colorings of

$$G_{n}$$.

Consequently,

$$P_{n}\left(k\right)=P\left(G_{n},k\right)$$

counts the number of feasible room assignments using

k

rooms.

**4.5.4 Interpretation of scheduling parameters**

Within the proposed two-period scheduling model, the parameter k denotes the number of available rooms and is therefore restricted to positive integer values. The feasibility of the scheduling problem is governed by the chromatic number of the conflict graph. Since

$$\chi \left(F_{n}\times P_{2}\right)=3,$$

the minimum feasible number of rooms in this model is

$$k_{\min}=3.$$

Thus, three rooms are necessary and sufficient to obtain a valid room assignment for every n≥2 .

2. The transition polynomial

$$\psi \left(k\right)=k^{4}-8k^{3}+26k^{2}-41k+26$$

plays a different role. By the recurrence relation $P_{n}\left(k\right)=\psi \left(k\right)P_{n-1}\left(k\right)$ , we have, for fixed k ,

$$\frac{P_{n}\left(k\right)}{P_{n-1}\left(k\right)}=\psi \left(k\right).$$

Hence, ψ(k) measures the multiplicative factor in the number of feasible schedules when one additional team is added, while the number of available rooms is kept fixed. For example,

ψ(3)=2,ψ(4)=22,ψ(5)=96.

Accordingly, with k=4 rooms fixed, increasing the model from n−1 to n teams multiplies the number of feasible schedules by 22. With k=5 rooms fixed, the corresponding multiplicative factor is 96.

For a fixed conference size n , the effect of changing the number of rooms is quantified by comparing the chromatic polynomial at different room numbers:

$$R_{n}\left(k_{2},k_{1}\right)=\frac{P_{n}\left(k_{2}\right)}{P_{n}\left(k_{1}\right)},\;k_{2}>k_{1}.$$

Thus, the recurrence factor ψ(k) describes scalability with respect to the number of teams for fixed k , whereas the ratio $R_{n}\left(k_{2},k_{1}\right)$ describes the change in scheduling flexibility obtained by increasing the number of available rooms for fixed n .

**4.5.5 Quantitative illustration**

We now illustrate the two distinct quantitative effects described above. The first concerns the growth in the number of feasible schedules when the number of teams increases while the number of rooms is fixed. The second concerns the change in scheduling flexibility when the number of rooms increases while the number of teams is fixed.

Scheduling with the minimum number of rooms ( k=3 ).

At the minimum feasible room count, we have ψ(3)=2 . By the recurrence relation,

$$P_{n}\left(3\right)=2P_{n-1}\left(3\right).$$

Thus, with three rooms, each additional team doubles the number of feasible schedules. Table 4 gives the corresponding values.

**Table 4.** Scheduling with the minimum number of rooms (k=3) .

Conference scale	Teams n	Valid schedules $P_{n}\left(3\right)$	Growth from previous n
Small conference	3	48	—
Medium conference	4	96	×2
Medium conference	5	192	×2
Large conference	6	384	×2

**Scalability with respect to the number of teams**

To illustrate the effect of adding one team while keeping the room count fixed, we compare $P_{5}\left(k\right)$ and $P_{4}\left(k\right)$ . The ratios are shown in Table 5, and they coincide exactly with ψ(k) .

**Table 5.** Scalability for fixed room availability.

Number of rooms k	$P_{4}\left(k\right)$	$P_{5}\left(k\right)$	$\frac{P_{5}\left(k\right)}{P_{4}\left(k\right)}$	Interpretation
3	96	192	ψ(3)=2	Adding one team doubles the schedules
4	2,811,072	61,843,584	ψ(4)=22	Adding one team multiplies schedules by 22
5	1,698,693,120	163,074,539,520	ψ(5)=96	Adding one team multiplies schedules by 96

The ratios in Table 5 compare $P_{5}\left(k\right)$ with $P_{4}\left(k\right)$ for the same value of k ; they therefore describe the effect of adding one team under fixed room availability. By contrast, the effect of increasing the number of rooms is measured by comparing $P_{n}\left(k\right)$ at different values of k for a fixed value of n .

**Effect of increasing the number of rooms**

For a fixed conference size n , the effect of increasing the number of available rooms is measured by comparing $P_{n}\left(k\right)$ at different values of k . Table 6 illustrates this effect for n=5 , using k=3 as the baseline.

**Table 6.** Effect of increasing the number of rooms for fixed n=5 .

Number of rooms k	$P_{5}\left(k\right)$	Flexibility ratio relative to k=3
3	192	1
4	61,843,584	322,102
5	163,074,539,520	849,346,560

For instance,

$$\frac{P_{5}\left(4\right)}{P_{5}\left(3\right)}=\frac{61,843,584}{192}=322,102.$$

Thus, for *n* = 5, increasing the number of rooms from

3

to

4

gives a flexibility ratio of

322,102

Similarly,

$$\frac{P_{5}\left(5\right)}{P_{5}\left(3\right)}=\frac{163,074,539,520}{192}=849,346,560.$$

In comparison, the value ψ(4)=22 is the fixed-room growth factor from $P_{4}\left(4\right)$ to $P_{5}\left(4\right)$ , namely

$$\frac{P_{5}\left(4\right)}{P_{4}\left(4\right)}=22.$$

**4.5.6 Summary of the Graph-Scheduling Correspondence**

The correspondence between the graph model and the scheduling interpretation is summarized in Table 7.

**Table 7.** From Graph Elements to Scheduling Interpretation.

Graph-theoretic element	Scheduling interpretation	Count	Meaning
$G_{n}=F_{n}\times P_{2}$	Two-period simplified scheduling model	—	Conflict graph for room assignment
$v_{0}^{A},v_{0}^{B}$	Coordinator sessions in periods A and B	2	Central scheduling sessions
$u_{i}^{A},w_{i}^{A}$	Two team-related sessions of team i in period A	2n	Team sessions in period A
$u_{i}^{B},w_{i}^{B}$	Two team-related sessions of team i in period B	2n	Team sessions in period B
All vertices	Sessions requiring rooms	4n+2	Total number of scheduled sessions
$v_{0}^{X}u_{i}^{X},v_{0}^{X}w_{i}^{X}$	Coordinator-team conflicts in period X	4n	These sessions cannot share a room
$u_{i}^{X}w_{i}^{X}$	Within-team conflict in period X	2n	The two team sessions require different rooms
$u_{i}^{A}u_{i}^{B},w_{i}^{A}w_{i}^{B}$	Cross-period team constraints	2n	Corresponding sessions cannot reuse the same room
$v_{0}^{A}v_{0}^{B}$	Coordinator cross-period constraint	1	Coordinator sessions cannot reuse the same room
All edges	Room-conflict constraints	8n+1	Total number of conflict constraints
Proper k -coloring	Valid room assignment using k rooms	$P_{n}\left(k\right)$	Counts all feasible schedules
$\chi \left(G_{n}\right)=3$	Minimum feasible number of rooms	3	Practical feasibility threshold
ψ(k)	Fixed- k team-growth factor	—	Growth factor when one team is added

**4.5.7 Model Limitations and Assumptions**

This scheduling interpretation is formulated under a controlled set of assumptions that make the correspondence between graph colorings and feasible room assignments explicit. It assumes that:

1. all teams have the same conflict structure;
2. all rooms are homogeneous and interchangeable;
3. the only constraints are those encoded by the edges of $F_{n}\times P_{2}$ ;
4. cross-period constraints require corresponding sessions to use different rooms;
5. no room capacities, time durations, preferences, or probabilistic constraints are included.

These assumptions delimit the scope of the model while preserving its role as a quantitative interpretation of the chromatic polynomial in a two-period resource-allocation setting.

## 5. Discussion

### 5.1. Theoretical contribution

This work provides an explicit algebraic characterization of the chromatic polynomial of $F_{n}\times P_{2}$ , a Cartesian product graph family with a layered triangular structure. The main result is the first-order recurrence $P_{n}\left(k\right)=\psi \left(k\right)P_{n-1}\left(k\right),\;n\;\geq \;3,$ together with the closed-form expression $P_{n}\left(k\right)={\left[\psi \left(k\right)\right]}^{n-2}P_{2}\left(k\right),\;n\;\geq \;2,$ where ψ(k) is the degree-four transition polynomial derived in Section 3.2.

The key structural reason for this formula is the conditional independence of the recursively attached block $B_{n}$ , as formalized in Lemma 3.1.1. Once the colors of the two central vertices are fixed and distinct, the four new peripheral vertices form a local extension problem whose number of admissible colorings depends only on k . Thus, the global chromatic polynomial is controlled by repeated copies of the same local transition count.

This result complements existing work on graph products and coloring ^6^ ^,^ ^7^ and on related structured families. ^8^ ^–^ ^12^ Compared with earlier Cartesian-product families such as $P_{2}\times P_{n}$ , $C_{3}\times P_{n}$ , $C_{3}□P_{n}$ , and $P_{3}□P_{n}$ , the present family has a different recursive mechanism: the parameter n increases the number of friendship triangles sharing a common center rather than extending a path direction. This shared-center structure reduces the chromatic recurrence to a single degree-four transition polynomial, reflecting the four new peripheral vertices added at each recursive step.

### 5.2 Structural, computational, and scheduling implications

The structural results show that

$$|V|=4n+2,\;|E|=8n+1,\;\chi \left(F_{n}\times P_{2}\right)=3.$$

Hence, the graph grows linearly in size while its chromatic number remains constant. This separates chromatic feasibility from enumerative growth: three colors are sufficient for all n≥2 , but the number of proper colorings grows according to the transition polynomial ψ(k) .

The closed-form expression also has a direct computational consequence. For fixed numerical k , the value $P_{n}\left(k\right)$ can be evaluated efficiently from the closed form using exponentiation by squaring in O(logn) arithmetic multiplications. This applies to fixed- k numerical evaluation, not to full symbolic expansion, since

$$\;\deg\;P_{n}\left(k\right)=4n+2.$$

Thus, the formula is both algebraically explicit and computationally useful for evaluating large members of the family.

The two-period scheduling interpretation gives a quantitative meaning to the same polynomial. In that model, $P_{n}\left(k\right)$ counts feasible room assignments under the stated conflict constraints. The chromatic number gives the minimum feasible number of rooms, $k_{\min}=3,$ while

$$\frac{P_{n}\left(k\right)}{P_{n-1}\left(k\right)}=\psi \left(k\right)$$

measures the growth in the number of feasible schedules when one additional team is added, and the number of rooms is kept fixed. The effect of increasing the number of rooms for fixed n is instead measured by ratios of the form $\frac{P_{n}\left(k_{2}\right)}{P_{n}\left(k_{1}\right)},k_{2}>k_{1}.$ This distinction gives the scheduling model a precise combinatorial interpretation while remaining within the stated scope of the model.

### 5.3 Limitations and future directions

The present work focuses on $F_{n}\times P_{2}$ . A natural extension is $F_{n}\times P_{m}$ for m>2 , which would correspond to a multi-period layered model and may require multiple boundary-coloring states rather than a single transition polynomial.

Another direction is to incorporate heterogeneous resources using list-coloring or weighted-coloring models. Such extensions could represent room capacities, team-specific requirements, or restricted availability of resources.

Finally, since the roots of $P_{n}\left(k\right)$ are contained in the union of the zero sets of $P_{2}\left(k\right)$ and ψ(k) , as described in Remark 4.3.5, this family may provide a tractable setting for studying chromatic-root multiplicities and their relation to partition-function phenomena such as the Potts model. ^16^

### Ethical considerations

This study does not involve human participants, animal subjects, or sensitive data. Therefore, no ethical approval was required.

### Use of AI-assisted technology

During manuscript revision, AI-assisted tools, including ChatGPT and DeepSeek, were used as supplementary aids for language editing, formatting support, and algebraic-verification checks. The authors critically reviewed all outputs and take full responsibility for the accuracy, originality, and integrity of the final manuscript.

## Data availability

This is a theoretical study in algebraic graph theory. All results, including the recurrence relation, the closed-form expression for the chromatic polynomial, and all numerical values, are derived analytically and presented within the article. No external datasets were generated or analyzed. All findings are fully reproducible using the formulas and methods provided in Sections 3 and 4.

### Reporting guidelines

This is a theoretical mathematical study and does not involve clinical trials, animal experiments, observational studies, or qualitative research. Therefore, no specific reporting guidelines (e.g., CONSORT, ARRIVE, STROBE, COREQ) are applicable.
