# Supplementary material for: Chromatic Polynomials of F n× P2  Graphs: Algebraic Analysis and Scheduling Applications
Source: F1000Res. 2026 Jun 12;15:351. Originally published 2026 Mar 4. [Version 2] doi: 10.12688/f1000research.176896.2 (PMC13560559; doi:10.12688/f1000research.176896.2)
Supplement: Peer review history

# Reviewer report: Reviewer response for version 2

**DOI:** 10.5256/f1000research.202763.r505470

**Published:** 2026-09-09

**Authors:** Julian D. Allagan

**Affiliations:** 1 Elizabeth City State University, Elizabeth City, USA; 2 Mathematics, University of Maryland Eastern Shore, Princess Anne, Maryland, USA

The revised manuscript is substantially improved, and the local counting argument for the transition polynomial appears correct. The main remaining issue is that the central formula can be simplified considerably; this affects the presentation of the principal theorem, the root analysis, and the claimed level of novelty.

 a. The main closed form should be replaced by the direct factorization. Lemma 3.1.1 already implies that, after choosing distinct colors for the two central vertices, the n attached blocks are conditionally independent and each contributes the same factor ψ(k). Hence the natural formula is P(F_n × P_2,k) = k(k−1)[ψ(k)]^n, with ψ(k) = k^4−8k^3+26k^2−41k+26. Indeed, the displayed polynomial P_2(k) factors exactly as k(k−1)[ψ(k)]^2. The present formula [ψ(k)]^(n−2)P_2(k) is correct, but it obscures the actual structure of the coloring count.

 b. The root and asymptotic sections should then be shortened and sharpened. Since ψ(k) = (k−2)(k^3−6k^2+14k−13), the complete root set of P_n is explicit: 0 and 1 occur with multiplicity one, while every root of ψ occurs with multiplicity n. Likewise, |P_n(k)|^(1/n) = |k(k−1)|^(1/n)|ψ(k)|, so the stated growth limit follows immediately for k ≠ 0,1, including roots of ψ. Corollary 4.3.4 is an exact ratio identity, not a genuine convergence result, and can be removed or stated simply as such.

 c. The scheduling interpretation still needs one modeling clarification. The manuscript calls the rooms homogeneous and interchangeable, whereas the chromatic polynomial counts assignments to k labeled colors. If rooms are intended to be indistinguishable, these counts overcount schedules by color permutations; otherwise, the rooms should be described as distinct resources of equal type. In addition, the vertical edges impose a cross-period room non-reuse constraint that is not standard in ordinary room scheduling and should be given a concrete operational justification.

 d. After the direct factorization above, most subsequent results become immediate consequences of one local extension count. This leaves the paper mathematically correct but fairly narrow. For a stronger graph-theory journal, the work would benefit from a genuinely new extension, for example F_n × P_m with m ≥ 3, where multiple boundary-color states lead to a nontrivial transfer matrix. Without such an extension, the paper is better positioned as a concise specialized note rather than a broad algebraic analysis.

 The central recurrence is sound, but the main theorem should be rewritten in its simplest exact form and the scheduling model clarified. After these corrections, it should be indexed.

Is the work clearly and accurately presented and does it cite the current literature?

Yes

If applicable, is the statistical analysis and its interpretation appropriate?

Not applicable

Are all the source data underlying the results available to ensure full reproducibility?

Partly

Is the study design appropriate and is the work technically sound?

Yes

Are the conclusions drawn adequately supported by the results?

Partly

Are sufficient details of methods and analysis provided to allow replication by others?

Partly

Reviewer Expertise:

Graph theory, combinatorics, functional analysis, machine learning and data science.

I confirm that I have read this submission and believe that I have an appropriate level of expertise to confirm that it is of an acceptable scientific standard, however I have significant reservations, as outlined above.

# Reviewer report: Reviewer response for version 2

**DOI:** 10.5256/f1000research.202763.r498725

**Published:** 2026-08-28

**Authors:** Muhammad Kamran Jamil

**Affiliations:** 1 Riphah International University, Islamabad, Islamabad Capital Territory, Pakistan

The manuscript presents an analytical study on the algebraic and enumerative properties of the chromatic polynomial for the Cartesian product graph family $F_n \times P_2$, where $F_n$ represents the friendship graph and $P_2$ denotes a two-vertex path. By implementing a recursive block decomposition along with a conditional independence framework, the authors establish a first-order recurrence relation and derive a closed-form polynomial expression governed by a single transition polynomial $\psi(k) = k^4 - 8k^3 + 26k^2 - 41k + 26$. Furthermore, the authors prove that the chromatic number is $\chi(F_n \times P_2) = 3$, analyze the real roots and asymptotic growth rate of the polynomial, validate the theoretical results through Mathematica computations, and provide an illustrative two-period scheduling model to demonstrate practical resource-allocation applications.

 Key Recommendations for Revision

- Revise the text to clearly differentiate between symbolic expansion complexity and fixed-$k$ numerical evaluation complexity using exponentiation by squaring $O(\log n)$, as the full symbolic polynomial degree grows linearly as $\text{deg}(\mathcal{P}_n(k)) = 4n + 2$.
- Ensure that the asymptotic growth rate formulation explicitly distinguishes between the general complex/real absolute-value expression $\lim_{n \to \infty} \vert{}\mathcal{P}_n(k)\vert{}^{1/n} = \vert{}\psi(k)\vert{}$ and the simplified positive integer coloring range $k \in \mathbb{Z}, k \ge 3$.
- Refine the scheduling application section to clearly emphasize its role as an illustrative, stylized conceptual model with explicitly stated assumptions (such as room homogeneity and pairwise conflict constraints) rather than a generalized scheduling solver.
- Double-check all mathematical typesetting, subscript alignments, and notation consistency across tables and text, specifically maintaining uniform usage of graph product symbols and local extension variables.
- Incorporate the following references into the literature review to provide broader context on chromatic invariants and graph domination properties:
  - refer to 1
  - refer to 2
  - https://doi.org/10.5923/j.am.20160602.01

Is the work clearly and accurately presented and does it cite the current literature?

No

If applicable, is the statistical analysis and its interpretation appropriate?

Not applicable

Are all the source data underlying the results available to ensure full reproducibility?

No source data required

Is the study design appropriate and is the work technically sound?

Yes

Are the conclusions drawn adequately supported by the results?

No

Are sufficient details of methods and analysis provided to allow replication by others?

Partly

Reviewer Expertise:

Graph Theory

I confirm that I have read this submission and believe that I have an appropriate level of expertise to confirm that it is of an acceptable scientific standard.

## References

[1] : Chromatic Zagreb indices for graphical embodiment of colour clusters. *Indonesian Journal of Combinatorics* .2019;3(1) : 10.19184/ijc.2019.3.1.6 10.19184/ijc.2019.3.1.6

[2] Johan Kok, Sudev Naduvath, Muhammad Kamran Jamil, Dominating Chromatic Weights Of Graphs, Applied Mathematics E-Notes, 18(2018), 51-61.

# Reviewer report: Reviewer response for version 2

**DOI:** 10.5256/f1000research.202763.r493245

**Published:** 2026-06-23

**Authors:** Siti Amiroch

**Affiliations:** 1 Universitas Islam Darul ‘ulum, Lamongan, Indonesia

I have reviewed this revised manuscript and have no further comments.

Is the work clearly and accurately presented and does it cite the current literature?

Partly

If applicable, is the statistical analysis and its interpretation appropriate?

Not applicable

Are all the source data underlying the results available to ensure full reproducibility?

No source data required

Is the study design appropriate and is the work technically sound?

Partly

Are the conclusions drawn adequately supported by the results?

Partly

Are sufficient details of methods and analysis provided to allow replication by others?

Partly

Reviewer Expertise:

NA

I confirm that I have read this submission and believe that I have an appropriate level of expertise to confirm that it is of an acceptable scientific standard.

# Reviewer report: Reviewer response for version 1

**DOI:** 10.5256/f1000research.195018.r474977

**Published:** 2026-05-11

**Authors:** Siti Amiroch

**Affiliations:** 1 Universitas Islam Darul ‘ulum, Lamongan, Indonesia

**Reviewer Report**

 The manuscript studies the chromatic polynomial of the graph family *F* *n* *x* *P* *2* , where *F* *n*  denotes the friendship graph and *P* *2*  is the path on two vertices. The topic is relevant to algebraic and combinatorial graph theory, and the recursive structure considered in the paper is potentially useful. The manuscript is generally organised in a logical sequence and the numerical examples help illustrate the proposed recurrence relation.

**General Evaluation**

 The main contribution of the paper is the derivation of a recurrence relation and a closed-form expression for the chromatic polynomial of *F* *n* *x* *P* *2* , together with structural observations, chromatic number analysis, asymptotic interpretation, and a scheduling application. The recursive approach is promising. However, several substantive issues require correction before the manuscript can be considered suitable for publication in a high-quality Scopus-indexed journal.

**Strengths**

 1. The paper addresses a specialised and relevant family of Cartesian product graphs.

 2. The recursive decomposition is a promising approach for obtaining a compact chromatic polynomial formula.

 3. The chromatic number result is useful and consistent with the triangular structure of the graph.

 4. The numerical examples support the recurrence formula for the tested cases.

 5. The attempt to connect the algebraic results with scheduling applications gives the paper potential applied relevance.

**Main Comments and Recommendations**

**1. Section 3.2 — Derivation of the transition polynomial**

 The derivation of the transition polynomial ψ(k) requires substantial clarification. The coefficients in

*ψ* *k* *=* *k* *4* *-8* *k* *3* *+26* *k* *2* *-41k+26* 

 are stated without a complete enumeration of the corresponding inclusion–exclusion terms. Since this polynomial is the central component of the recurrence and closed-form formula, the manuscript should provide either a full counting table, a structured case analysis, or an appendix containing the complete derivation.

**2. Section 4.1 — Claim on non-planarity**

 The assertion that *F* *n* *x* *P* *2*  is non-planar for n ≥ 3 is not sufficiently justified. The manuscript states that the graph contains a *K* *3,3*  minor, but no explicit construction of such a minor is provided. This is a substantial mathematical claim and should either be proved rigorously by specifying the required branch sets or revised if the claim does not hold.

**3. Section 4.2 — Computational complexity**

 The statement that the closed-form expression reduces the computation of *P* *n* *(k)*  to O(log n) is too broad. This claim is valid only for numerical evaluation at a fixed value of k using exponentiation by squaring. It does not apply to symbolic expansion of the polynomial, since the degree and output size increase with n. The statement should be qualified accordingly.

**4. Section 4.3 — Asymptotic growth**

 The asymptotic statement involving *P* *n* *(k)* *1* *n*   should be formulated more carefully. For general real or complex values of k, the mathematically safer formulation should involve *P* *n* *(k)* *1* *n* . The current expression is valid only under additional restrictions, for example for integer k ≥ 3. These restrictions should be made explicit.

**5. Section 4.5 — Scheduling interpretation**

 The scheduling interpretation contains two important issues. First, the non-integer real root of ψ(k) should not be interpreted as a theoretical minimum number of rooms. In a scheduling problem, the number of rooms is discrete, and feasibility is determined by the chromatic number χ = 3. Secondly, ψ(4) = 22 is interpreted as the gain obtained by increasing the number of rooms from 3 to 4, whereas ψ(k) represents the growth factor when the number of teams increases while k is fixed. These interpretations should be corrected to avoid overstating the practical implications of the model.

**6. Section 4.5 — Mapping between the graph and the scheduling model**

 The correspondence between *F* *n* *x* *P* *2*  and the proposed conference scheduling model is not yet sufficiently precise. The graph has 4n + 2 vertices, but the description involving one coordinator, n teams, two periods, and two tasks per team does not clearly explain how these scheduling entities produce exactly 4n + 2 vertices. A clearer vertex-to-session and edge-to-conflict mapping is required for the application to be convincing.

**Final Recommendation**

 The manuscript has a promising mathematical core, particularly in its recursive treatment of *F* *n* *x* *P* *2* . However, the derivation of the transition polynomial, the structural claim on non-planarity, the asymptotic formulation, and the scheduling interpretation require substantial revision. In its present form, the manuscript is not yet suitable for publication in the journal. **A major revision is recommended.**

Is the work clearly and accurately presented and does it cite the current literature?

Partly

If applicable, is the statistical analysis and its interpretation appropriate?

Not applicable

Are all the source data underlying the results available to ensure full reproducibility?

No source data required

Is the study design appropriate and is the work technically sound?

Partly

Are the conclusions drawn adequately supported by the results?

Partly

Are sufficient details of methods and analysis provided to allow replication by others?

Partly

Reviewer Expertise:

NA

I confirm that I have read this submission and believe that I have an appropriate level of expertise to confirm that it is of an acceptable scientific standard, however I have significant reservations, as outlined above.

# Response

**Published:** 2026-05-28

**Authors:** Sarah M. Talab

**Affiliations:** Mathematics, Tikrit University, Tikrit, Saladin Governorate, Iraq

We sincerely thank the reviewer for the careful evaluation and constructive comments. We appreciate the reviewer’s recognition of the relevance of the topic, the recursive approach, the chromatic number result, the numerical verification, and the potential scheduling interpretation. We have revised the manuscript substantially to address all points raised.

**Comment 1. Derivation of the transition polynomial.**

 We agree that the derivation of ψ(k) required more detail. In the revised manuscript, Section 3.2 now includes a structured combinatorial case analysis for the local extension count Ndiff(k). After fixing the colors of the two central vertices, we count the admissible colorings of the four new peripheral vertices and obtain

 Ndiff(k)=2(k−2)^3+(k−2)(k−3)(k^2−5k+7)=ψ(k).This gives

 ψ(k)=k^4−8k^3+26k^2−41k+26.

 A summary table of the enumeration has also been added.

**Comment 2. Non-planarity claim.**

 We agree that the previous non-planarity statement required a rigorous proof. To avoid unsupported claims outside the main focus of the paper, we removed this assertion from the revised manuscript. The structural analysis now focuses on the order, size, connectivity, and degree sequence, which are directly used in the chromatic analysis.

**Comment 3. Computational complexity.**

 We revised the complexity statement. The manuscript now states explicitly that the O(log⁡ n) complexity applies only to fixed-k numerical evaluation using exponentiation by squaring. It does not apply to full symbolic expansion, since

 deg⁡Pn(k)=4n+2.

**Comment 4. Asymptotic growth.**

 We reformulated the asymptotic result more carefully. For fixed k with P2(k)≠0, the revised theorem states

 limn→∞∣Pn​(k)∣^1/n=∣ψ(k)∣

 For integer k≥3, where Pn(k) and ψ(k) are positive, this reduces to

 lim⁡n→∞Pn(k)^1/n=ψ(k)..

**Comment 5. Scheduling interpretation.**

 We revised the scheduling section to correct the interpretation. The non-integer real root of ψ(k) is no longer interpreted as a minimum number of rooms. The minimum feasible number of rooms is now stated to be determined by the chromatic number:

 kmin⁡=3.

 We also clarified that ψ(k) measures the growth factor when one additional team is added while the number of rooms is fixed. The effect of increasing the number of rooms is now measured separately by ratios of the form

 Pn(k2)/Pn(k1),k2>k1​.

**Comment 6. Mapping between the graph and the scheduling model.**

 We expanded the scheduling model by adding a precise vertex-to-session and edge-to-conflict mapping. The revised version explains that the two central vertices represent coordinator sessions in the two periods, while the 4n peripheral vertices represent two team-related sessions for each of the n teams in each of the two periods. Hence the model has exactly

 2+4n=4n+2

 sessions, matching the vertex count of Fn×P2​. The edge constraints are also described explicitly.

 We thank the reviewer again for the detailed comments. We believe that the revised manuscript is now more rigorous, clearer, and more precise in its mathematical derivation and scheduling interpretation.

# Reviewer report: Reviewer response for version 1

**DOI:** 10.5256/f1000research.195018.r476650

**Published:** 2026-04-28

**Authors:** Wafiq Hibi

**Affiliations:** 1 Academic College of Sakhni, Sakhnin, Israel

The manuscript studies the chromatic polynomial of the graph family F n × P 2 ​, where Fn denotes the friendship graph and P2​ is the path on two vertices. The topic is relevant to algebraic and combinatorial graph theory, and the paper addresses a graph family that has received limited direct attention in the literature. The manuscript is generally well organized and the results are presented in a logical sequence.

**General Evaluation:**

 The main contribution of the paper is the derivation of a recurrence relation and a closed-form expression for the chromatic polynomial of F n × P 2​, together with structural properties, chromatic number analysis, asymptotic interpretation, and an illustrative scheduling application. The central formula appears mathematically consistent, and the numerical examples support the stated recurrence. The overall direction of the work is sound and potentially useful for researchers interested in graph products and chromatic invariants.

**Strengths**

1. The paper studies a nontrivial and specialized family of Cartesian product graphs.
2. The recurrence framework is elegant and gives a compact closed-form representation.
3. The structural results (order, size, chromatic number) are useful and coherent.
4. Numerical verification is included and supports the theoretical formulas.
5. The scheduling section provides a practical interpretation of the graph-coloring model.

**Main Comments and Recommendations**

**1. Proof of the Transition Polynomial**

 The most important result is the derivation of the transition polynomial

 ψ(k)= = k 4 − 8 k 3 + 26 k 2 − 41 k + 26

 Although the result appears correct, the derivation is presented too briefly. The manuscript states that Inclusion–Exclusion is applied to eight constraints, but the key coefficients (26, 41, 26) are not derived transparently. For mathematical clarity and reproducibility, the authors should expand this section substantially.

**Recommendation:** Include either:

- a complete combinatorial derivation, or
- a structured case analysis, or
- a supplementary appendix containing the full counting argument.

**2. Recursive Independence Argument**

 The argument that the newly attached block contributes independently once the colors of the two central vertices are fixed is reasonable, but it would benefit from a more formal proof and clearer notation.

**Recommendation:** State the independence lemma more explicitly and clarify why no additional constraints arise from previous blocks.

**3. Notation and Typography**

 Several expressions suffer from formatting inconsistencies (subscripts, spacing, symbols, repeated notation, typographical artifacts). This occasionally affects readability.

**Recommendation:** Carefully revise notation throughout the manuscript and ensure all formulas are typeset consistently.

**4. Scheduling Application**

 The application is interesting as a motivating example, but it should be presented clearly as an illustrative use-case rather than a major applied breakthrough.

**Recommendation:** Shorten slightly or explicitly frame it as a demonstration of potential applicability.

**Final Recommendation**

 This is a worthwhile mathematical contribution with a correct and interesting central result. However, the exposition of the main proof should be strengthened before final acceptance. After moderate revision focused on rigor, clarity, and presentation, the paper would be suitable for indexing.

Is the work clearly and accurately presented and does it cite the current literature?

Partly

If applicable, is the statistical analysis and its interpretation appropriate?

Not applicable

Are all the source data underlying the results available to ensure full reproducibility?

No source data required

Is the study design appropriate and is the work technically sound?

Yes

Are the conclusions drawn adequately supported by the results?

Yes

Are sufficient details of methods and analysis provided to allow replication by others?

Partly

Reviewer Expertise:

Mathematics Education and Applied Graph Theory in Teaching and Learning Contexts

I confirm that I have read this submission and believe that I have an appropriate level of expertise to confirm that it is of an acceptable scientific standard, however I have significant reservations, as outlined above.

## References

[1] Hibi, W. (2022). Assembling Planer Graphs to Service the Coloring Number. Review of International Geographical Education Online, 12(1), 28-31.

# Response

**Published:** 2026-05-28

**Authors:** Sarah M. Talab

**Affiliations:** Mathematics, Tikrit University, Tikrit, Saladin Governorate, Iraq

We sincerely thank the reviewer for the careful reading of the manuscript and for the constructive comments. We appreciate the positive assessment of the topic, the recurrence framework, the structural results, the numerical verification, and the scheduling interpretation. We have revised the manuscript to improve rigor, clarity, notation, and presentation.

**Comment 1. Proof of the transition polynomial.**

 We agree that the derivation of

 ψ(k)=k^4−8k^3+26k^2−41k+26\

 needed to be expanded. In the revised manuscript, Section 3.2 now contains a structured combinatorial case analysis for the local extension count Ndiff(k). After fixing the colors of the two central vertices, we count the admissible colorings of the four new peripheral vertices and obtain

 Ndiff(k)=2(k−2)^3+(k−2)(k−3)(k^2−5k+7)=ψ(k).

 A summary table of the case analysis was also added.

**Comment 2. Recursive independence argument.**

 We added Lemma 3.1.1 to formalize the conditional independence of the newly attached block Bn​. The lemma clarifies that the new peripheral vertices have no edges to peripheral vertices of earlier blocks; hence, once the two central colors are fixed, all remaining constraints are local to Bn​. This justifies the recurrence.

**Comment 3. Notation and typography.**

 We revised the manuscript for notational consistency, subscripts, spacing, formula formatting, table presentation, and typographical artifacts. We also clarified that × denotes the Cartesian product throughout the paper.

**Comment 4. Scheduling application.**

 We revised the scheduling section as an illustrative two-period model. The revised version includes explicit vertex-to-session and edge-to-conflict mappings, clarifies that χ(Fn×P2)=3 gives the minimum feasible number of rooms, and interprets ψ(k) correctly as the fixed-room team-growth factor rather than the effect of adding rooms. The assumptions and limitations of the model were also stated explicitly.

 We thank the reviewer again for the helpful comments. We believe the revised version now provides a clearer derivation, a stronger recurrence argument, and a more precise interpretation of the scheduling application.
